# Damage-associated molecular patterns (DAMPs) in diseases: implications for therapy

**DOI:** 10.1186/s43556-025-00305-3

**Published:** 2025-08-29

**Authors:** Heya Lin, Wei Xiong, Lili Fu, Jie Yi, Jiantang Yang

**Affiliations:** School of Stomatology, Zunyi Medical University, Zunyi, Guizhou China

**Keywords:** Damage-associated molecular patterns, Pattern recognition receptors, Pathways, Therapeutic strategies, Immune response

## Abstract

Damage-associated molecular patterns (DAMPs) are endogenous danger signal molecules released by damaged, stressed or dead cells that bind to pattern recognition receptors (PRRs), activating immune responses and inflammatory signaling pathways to play critical regulatory roles in various pathophysiological processes. This review classifies DAMPs into three major categories (protein-based, nucleic acid-based and mitochondria-derived) based on distinct molecular characteristics and biological functions, analyzing their structural features and functional differences. We systematically summarize current understanding of DAMP molecular transformation mechanisms, release pathways and recognition processes, with in-depth discussion of their pathological roles in major diseases including cancer, cardiovascular diseases and respiratory disorders. Particular emphasis is placed on the molecular recognition mechanisms between DAMPs and PRRs (TLRs, NLRs, CLRs and RAGE), and the disease regulatory networks formed by activated key signaling pathways (NF-κB, MAPK, inflammasomes and cGAS-STING). Current DAMP/PRR-targeted therapeutic strategies are comprehensively reviewed, including: modulating cell death pathways to reduce DAMP release, neutralizing DAMP activity using monoclonal antibodies, developing small-molecule inhibitors to block signaling pathways, and employing enzymatic degradation or gene silencing technologies for precise intervention. While showing promise in inflammatory and cancer disease models, these approaches face clinical translation challenges including DAMP molecular heterogeneity, inefficient drug delivery systems, and the complexity of multi-target synergistic mechanisms. Potential solutions involving nanoparticle delivery systems, AI-driven personalized treatment optimization and gene editing technologies are discussed. This review aims to provide references for developing novel therapeutics targeting the DAMP/PRR signaling axis, potentially opening new treatment avenues for cancer, neurodegenerative diseases, cardiovascular diseases and inflammatory disorders.

## Introduction

**Fig. 1:**
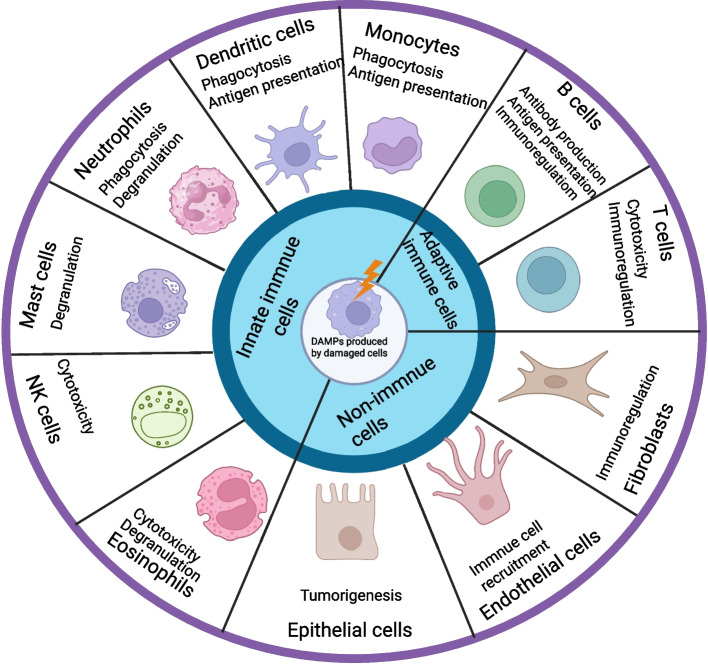
The source of DAMP. DAMP is mainly derived from innate immune cells including monocytes, macrophages, dendritic cells, neutrophils, mast cells, natural killer cells, eosinophils and others. Adaptive immune cells, including B cells and T cells. Non-immune cells, including endothelial cells, epithelial cells and fibroblasts, etc. DAMP, as a signalling molecule, interacts with these cells and plays an important role in initiating the immune response and regulating the immune response, thus bridging the gap between cellular damage and immune response

The meticulous regulation of the immune system has long revolved around the core tenet of "self—recognition," initially put forward by Charles Janeway in 1989. Janeway demonstrated that the immune system identifies pathogen—associated molecular patterns (PAMPs) via germline—encoded pattern—recognition receptors (PRRs), thus triggering anti—infective defense mechanisms [1]. This classical paradigm, founded on"non—self"recognition, offers the molecular foundation for comprehending infectious inflammation. Nevertheless, the "Danger Theory," put forward by Polly Matzinger in 1994, contends that the fundamental impetus for immune activation is not solely the discrimination between self and non—self, but rather the detection of danger signals, encompassing bacterial toxins, viruses, rapidly proliferating tumor cells, and non—programmed (necrotic) cell death [2]. This theory presents a novel explanatory framework for sterile inflammation. Building upon this, Walter Land put forward the concept of "damage-associated molecular patterns" (DAMPs) in 2003 to differentiate endogenous danger signals from traditional pathogen-derived signals. Subsequent to the discovery of numerous endogenous pro-inflammatory molecules, Seong and Matzinger systematized the dual functions of DAMPs in 2004: they convey alarm signals initiated by exposed hydrophobic fractions and regulate both innate and adaptive immune responses [3] (Fig. 1).

Research has demonstrated that DAMPs play pivotal roles in various diseases, including cancer, cardiovascular disorders, and pulmonary diseases, making them promising therapeutic targets. However, despite over two decades of investigation elucidating their complex biological functions in disease progression, DAMP-targeted therapies remain in the early translational phase. Current interventions are predominantly confined to animal model validation, with limited clinical translation due to several challenges: inadequate molecular targeting efficiency, suboptimal delivery system performance, and unresolved multi-target synergistic mechanisms.

In this context, we summarize the mechanism of conversion of endogenous molecules to DAMPs, their release pathways, classification systems, recognition modes, and their pathological roles in various diseases, and explain in detail current therapeutic strategies for DAMPs/PRRs, including cell death pathway modulation, monoclonal antibody interventions, signaling pathway inhibition, and gene silencing technologies, and discuss the challenges of these therapeutic strategies. At the same time, the challenges and potential solutions of these therapeutic strategies are discussed, including novel delivery systems such as nanoparticles, gene editing technologies, and AI-driven individualized protocols. In this review, we have taken"basic molecular mechanism—pathological network—therapeutic strategy—future challenges"as the logical thread, which runs through the whole chain of DAMPs research and builds up a systematic framework, aiming to provide a reference for subsequent researchers.

## Mechanisms and release pathways of endogenous molecules into DAMPs

### Conversion pathways of endogenous molecules to DAMPs

Under physiological homeostasis, host endogenous molecules typically maintain an immunologically quiescent state. Conversely, pathological stimuli, including infection and trauma, can trigger their transformation into DAMPs via multiple mechanisms.

First and foremost, the most prevalent transformation transpires when intracellular molecules enter the extracellular realm as a result of the disruption of physical barriers. The release mechanism can be classified into two primary categories: passive release via cell death or active secretion by viable cells under stress. Once DAMPs emerge in the extracellular milieu, they can initiate a cascade of downstream stress—response mechanisms by binding to evolutionarily conserved PRRs. For instance, PAUF/API5 secreted by tumor cells activates TLR2/4 to promote dendritic cell maturation [4, 5], and mitochondrial DNA(mtDNA) released from mitochondria induces interferon production via the cGAS-STING pathway [6].

Secondly, certain DAMPs demonstrate concentration-dependent pro-inflammatory functions, rendering concentration imbalance another crucial mechanism for DAMP transformation. Research findings have indicated that, in comparison to cells in a homeostatic state, upregulated proteoglycans binding to CD14/CD44 activate TLR2/4 in damaged cells [7]; During metabolic disorders, the accumulation of fatty acids activates the NLRP3 inflammasome via the PERK/eIF2αpathway [8]; While potassium ion efflux [9, 10] or CLIC-mediated Cl- efflux triggers NLRP3 assembly [11].

Furthermore, alterations in the chemical or physical properties of endogenous molecules constitute another significant pathway for the conversion of DAMPs. At the chemical level, molecular degradation, misfolding, or post-translational modifications can convert these molecules into pro-inflammatory ones. For instance, the degradation products of high-molecular-weight hyaluronic acid activate TLR2/4 and cluster of differentiation 44 (CD44), thereby promoting the inflammatory process in obesity and rheumatoid arthritis (RA) [12]. Changes in physical properties can cause lysosomal disruption through mechanical forces generated by crystals, leading to NLRP3 inflammasome activation. This mechanism explains how urate crystal deposition in joints induces gout [13] and cholesterol crystals in vascular walls promote atherosclerosis [14].

### Release mechanisms for DAMPs

**Fig. 2:**
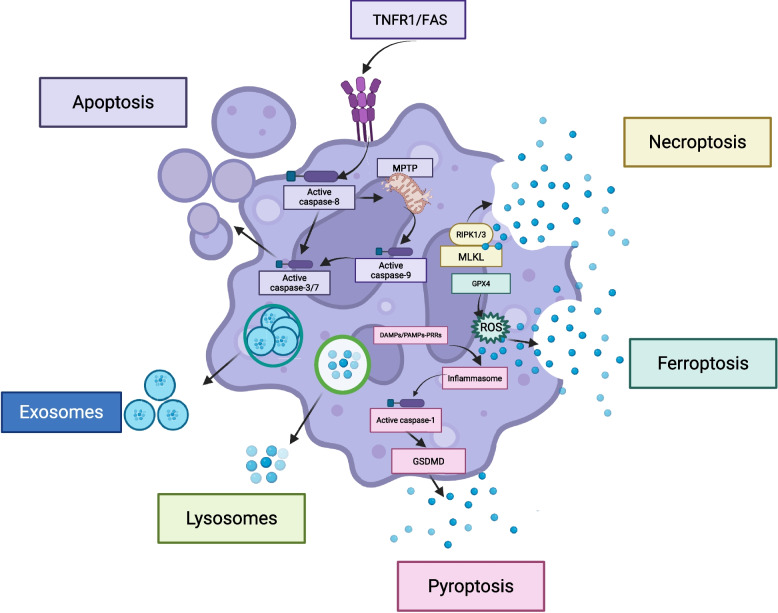
The release mechanisms of DAMPs. The different release mechanisms of DAMPs can be broadly classified into two categories:passive release mainly due to cell death and active release from living cells represented by cytotoxicity. Cell death modes include necrosis, pyroptosis, apoptosis, and Ferroptosis

The release mechanisms of DAMPs can be generally classified into two categories: passive release mainly caused by cell death and active release from living cells, exemplified by exocytosis. Cell death modalities encompass necrosis, pyroptosis, apoptosis, and ferroptosis. It is noteworthy that in the context of passive DAMP release, apart from necrosis, other forms of cell death are not entirely passive but rather regulated processes (Fig. 2).

#### Passive release

Severe chemical or physical stimuli, along with tissue ischemia and hypoxia, can induce cellular necrosis. This phenomenon is manifested by cellular swelling, plasma membrane rupture, and eventually the disappearance of cellular boundaries. Historically, necrosis has been regarded as a "passive" process. However, recent research has uncovered regulatory pathways for specific necrotic events. These involve the initiation of necrosome activation mediated by RIPK1/RIPK3 and the formation of 4 nm inner—diameter cation channels via MLKL phosphorylation—oligomerization, which enhances plasma membrane permeability and promotes the release of DAMPs [15–18]. Numerous DAMPs have been demonstrated to be released through necrotic processes, including HMGB1 (High Mobility Group Box 1 Protein), ATP, exRNA, cell-free DNA(cfDNA), eCIRP, histones, and heat shock proteins (HSPs) [19–23].

Pyroptosis represents a caspase-dependent programmed cell death, which is typified by a core pathway. In this pathway, the recognition of PAMPs or DAMPs by PRRs activates inflammasomes (e.g., NLRP3), thereby initiating the activation of inflammatory caspases (caspase-1/−4/−5/−11) [24, 25]. These activated caspases specifically cleave proteins of the Gasdermin (GSDM) family, thereby liberating their N—terminal domains. These liberated N—terminal domains oligomerize to form plasma membrane pores with a diameter of 10—18 nm, facilitating the release of small molecules such as IL—1β and IL—18 [25]. In contrast to the cation—selective pores observed in necrosis, pyroptotic pores display non—selective permeability. This characteristic leads to negligible alterations in osmotic pressure and culminates in cell death that is independent of cellular lysis [26]. Notably, the release of DAMPs during pyroptosis is temporally regulated and selective. In the early stages, caspase—1—mediated cleavage activates IL—1β and IL—18 for secretion via GSDM pores. In contrast, in the late stages, membrane rupture promotes the release of macromolecular DAMPs such as HMGB1, ATP, and cfDNA [19, 27–31].

Apoptosis, a programmed cell death process predominantly mediated by caspase cascade reactions, is distinguished by two crucial characteristics: the preservation of plasma membrane integrity and the formation of apoptotic bodies. This process is regulated through the coordinated interplay of extrinsic and intrinsic pathways.In the extrinsic pathway, ligands (TNF—α/FasL) bind to trimeric death receptors (TNFR1/Fas) and recruit the Fas-associated death domain protein (FADD) adaptor proteins along with pro—caspase—8/—10 via death domains (DD), thereby forming the death—inducing signaling complex (DISC). This complex enables the auto—proteolytic activation of initiator caspase—8. Subsequently, the activated caspase—8 either directly cleaves effector caspases—3/—7 or initiates the intrinsic pathway through the cleavage of the BH3—only protein Bid (a member of the Bcl—2 family).The intrinsic pathway entails the insertion of Bid into the mitochondrial outer membrane, which promotes the oligomerization of Bax/Bak and the subsequent formation of mitochondrial permeability transition pores (mPTP). These pores facilitate the release of cytochrome c into the cytosol. Cytochrome c binds to Apaf—1 and dATP to form apoptosomes, which in turn activate caspase—9 and downstream effector caspases. Both pathways ultimately converge on effector caspases—3/—6/—7, which execute apoptotic changes through the proteolytic cleavage of key cellular components, including Lamin, PARP, and cytoskeletal proteins (Actin/Tubulin). These cleavage events induce characteristic morphological alterations, such as chromatin margination, nuclear condensation, and plasma membrane blebbing [32].

Ferroptosis, an iron-dependent modality of programmed cell death, is distinguished by the accrual of lipid peroxides and the subsequent rupture of the plasma membrane. Morphologically, it is manifested as cellular swelling, organelle enlargement, and moderate chromatin condensation [33]. The process is initiated by intracellular iron overload triggered by either iron chelators (DFO) or GPX4 inhibitors (RSL3). The accumulated iron generates reactive oxygen species (ROS) through the Fenton reaction, which then activates lipoxygenases (LOXs) to catalyze the metabolism of arachidonic acid. This metabolic cascade yields lipid oxidation products such as 4HNE and oxPLs [33, 34].Glutathione peroxidase 4 (GPX4), a pivotal antioxidant enzyme, functions as the core regulator of ferroptosis through the suppression of lipid peroxidation. Therefore, the pharmacological inhibition of GPX4 activity alleviates this suppressive effect and constitutes a primary mechanism for inducing ferroptosis [35].

#### Active release

Apart from being released through diverse cell death pathways, DAMPs can also be actively secreted by viable cells via secretory lysosomes and exosomes. During lysosomal exocytosis, the stimulation of cell—surface receptors induces an elevation in the intracellular Ca^2+^concentration. Synaptophysin (Syt) mediates the translocation of lysosomes towards the microtubule—organizing center (MTOC), and subsequently, they are transported to the secretion sites via motor proteins and actin filaments. The docking and fusion with the plasma membrane are mediated by the RAB—SNARE complex [36], releasing DAMPs such as HMGB1, ATP, and eCIRP. This process is significantly enhanced under stress conditions [29, 37, 38].

Exosomes, which originate as multivesicular bodies (MVBs) formed through endosomal budding [39], undergo biogenesis regulated by the ESCRT complex and associated proteins including VPS4, VTA1, and ALIX [40]. These vesicles are conveyed to the plasma membrane through the coordinated actions of actin filaments, microtubules, and RAB proteins, and are ultimately discharged via the RAB—SNARE complex. Exosomes carry diverse DAMPs such as HMGB1, ATP, exRNA, cfDNA, and HSPs, which play crucial roles in intercellular communication [41–43].

## Classification of DAMPs

### Protein-based DAMPs

#### HMGB-1

HMGB1, among the most comprehensively characterized DAMPs, is a 215-amino acid non-histone nuclear protein that consists of three structural domains: two tandem high-mobility group (HMG) box domains (Box A and Box B) and a 30-amino acid-rich C-terminal tail [44]. The Box A and Box B domains play a role in mediating DNA binding, whereas the C—terminal tail engages in an interaction with histones and directly modulates the binding specificity of HMGB1 to DNA via its interaction with the N—terminal box domains. HMGB1 encompasses two nuclear localization signal sequences (NLSs) that are respectively situated between the A/B boxes and the C—terminal tail. These NLSs contribute to chromosomal anchoring and the formation of histone complexes, thereby stabilizing the nucleosome structure. By inducing DNA bending, HMGB1 exposes transcription factor binding sites (such as those for p53 and NF -κB), consequently participating in the processes of gene transcription regulation and DNA repair [45–47].

**Fig. 3:**
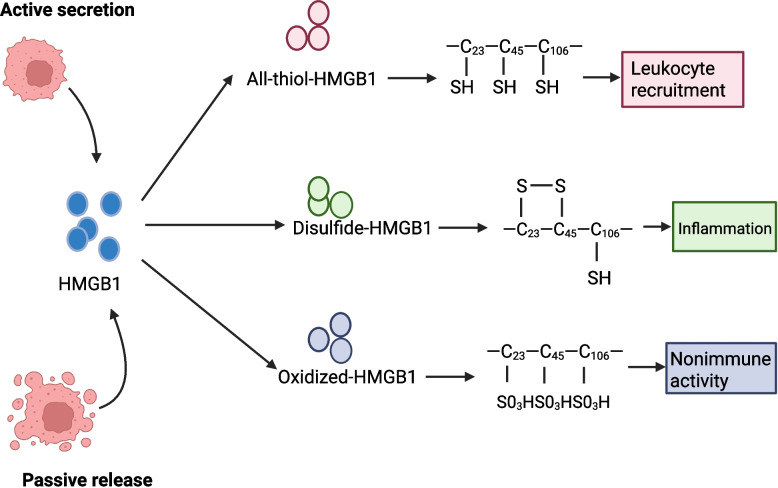
Redox states of HMGB1. The three redox states of HMGB1 differ in structure and activity. The reduced form contains sulfhydryl groups on all three cysteine residues, and it is this form that is able to bind to RAGE and activate inflammatory pathways. The disulfide form contains a disulfide bond between the cysteine residues at positions 23 and 45, and this bond increases its stability. The final inactivated form is known as the oxidised form and has sulphonated cysteines at all three positions

The extracellular function of HMGB1 is determined by the redox states of three cysteine residues (C23, C45, and C106) in its DNA-binding domain and acidic tail [48]. Via redox-dependent receptor recognition, HMGB1 demonstrates dual immunomodulatory effects, manifesting both pro-inflammatory and immunosuppressive activities. The fully reduced HMGB1 (with all cysteines in the thiol state) serves as a damage signal that recruits monocytes and leukocytes. This form forms a complex with CXCL12 to initiate leukocyte recruitment through CXCR4 interaction [49], while also activating the NF-κB pathway through TLR2/4/9 to induce pro-inflammatory cytokine (IL-6, TNF-α) secretion from monocytes. The disulfide isoform (C23-C45 disulfide bond with C106 thiol) binds macrophage TLR4, triggering production of pro-inflammatory cytokines including TNF-α, IL-1, and type 1 interferon [50]. This form is capable of undergoing redox conversion in vivo, specifically being reduced to the all—thiol state or further oxidized to sulfonated HMGB1 by ROS. The fully oxidized (sulfonate) HMGB1 predominantly mediates immunosuppression during apoptosis via the engagement of the receptor for advanced glycation end—products (RAGE) [51] (Fig. 3).

Specifically, pretreatment with HMGB1 diminishes the production of TNF-α induced by LPS in macrophages and attenuates phagocytic activity through the inhibition of the NF-κB and MAPK/JNK pathways [52–54]. Neutrophils pretreated with HMGB1 exhibit a notably diminished generation of ROS induced by phorbol 12-myristate 13-acetate (PMA). Moreover, clinical correlation analysis indicates a significant negative correlation between plasma HMGB1 levels and neutrophil ROS production in patients with septic shock [55]. Regarding the regulation of T cell polarization, HMGB1 induces the shift of CD4^+^ T cells towards the Th2 phenotype, resulting in a reduction in IFN—γ secretion and facilitating Treg proliferation. Anti—RAGE antibodies can effectively reverse Th2 polarization [56, 57]. Myeloid-derived suppressor cells (MDSCs) impede both innate and adaptive immune responses via multiple mechanisms. Moreover, HMGB1 may facilitate post—injury systemic immunosuppression by augmenting the expansion of MDSCs [58, 59]. Research findings have indicated that HMGB1 prompts the differentiation, survival, migration, and activation of MDSCs through the RAGE-STAT3 pathway, and pretreatment with anti-HMGB1 antibody notably suppresses the infiltration of MDSCs [60, 61].

#### HSP

HSPs, a family of molecular chaperones that are evolutionarily conserved, participate in protein folding, stabilization, and transport via binding to hydrophobically exposed substrate proteins through the AHA structural domain [62]. These proteins are synthesized by diverse cell types upon induction by stress factors, encompassing both low and high temperatures, ultraviolet radiation, pathogens, and other stress modalities [63, 64]. Under stress conditions, the heat shock response is fundamentally a cytoprotective mechanism characterized by the selective and elevated expression of HSPs. This mechanism facilitates cell survival in the face of oxidative and hyperthermic challenges by augmenting protein folding efficiency and suppressing apoptosis [65]. The specific up—regulation of HSPs in response to heat shock validates their pivotal role in preserving protein homeostasis via molecular chaperone functions. Consequently, this up—regulation augments cellular resistance to stress and facilitates environmental adaptation.

As DAMPs, the aberrant extracellular accumulation of HSPs is intricately correlated with chronic inflammatory disorders, including RA and atherosclerosis, which exhibit high immunogenicity [66]. Research findings have indicated that HSP70 activates the NF-κB pathway via the TLR2/4—CD14 receptor complex, prompting monocytes to secrete cytokines including TNF-αand IL-6. Simultaneously, HSP90 binds to the CD91 receptor and upregulates MHC—II molecules as well as co—stimulatory molecules CD80/CD86 in dendritic cells (DCs), promoting their transition from an immature to an antigen—presenting phenotype [66]. In patients with polytrauma, the serum concentrations of HSP70 are 10 times higher immediately post—injury compared to those in the control group. Moreover, the decline of these concentrations within 60 to 90 h indicates an improved prognosis [65]. However, in sepsis, although the HSP family is generally elevated (HSP27/60/70/90) [67, 68], there are significant differences in their subtype-specific functions. For example, in a mouse model of inflammatory sepsis-induced acute lung injury (ALI), HSP90 inhibitors significantly reduced mortality and improved lung function, as well as local and systemic inflammation [69]. In contrast, HSP70.1/3 knockout mice demonstrated elevated levels of TNF-αand IL-6 in lung tissue, accompanied by a higher mortality rate following cecal ligation and puncture (CLP). Mechanistically, HSP70 diminishes the transcription of pro—inflammatory cytokines by directly binding to inhibit the Ser^536^ phosphorylation and nuclear translocation of the NF -κB p65 subunit [70]. Other investigations have demonstrated that in cerebral ischemia, neurodegenerative disorders, and epilepsy, HSP70 suppresses abnormal protein aggregation and apoptosis, diminishes the formation of intracellular inclusion bodies, and enhances neurological prognosis [71–73]. Thus, accumulating evidence suggests that HSPs exhibit a dual immunomodulatory effect.

#### S100 protein

The S100 proteins constitute a family encompassing at least 25 low-molecular-weight calcium-binding proteins situated within the cytoplasm of leukocytes, microglia, and astrocytes. These proteins participate in intracellular processes such as cell proliferation, oxidative stress regulation, and DNA repair [63, 74]. These proteins have been associated extracellularly with cancer, inflammatory disorders, autoimmune diseases, or cardiovascular diseases (CVD) and have been detected in diverse body fluids, including serum, urine, sputum, cerebrospinal fluid, and feces, from patients with these conditions [74–79]. S100 proteins initiate the p38 MAPK and ERK1/2 kinase cascades, along with activating NF-κB transcription factors, via ligand—receptor interactions with RAGE, TLR4, and other receptors. Consequently, these proteins mediate the expression of NF-κB transcription factors and their pro—inflammatory effects [80–84]. In a myocardial infarction research, it was discovered that the passive release of S100A1 from necrotic cardiomyocytes reached its peak at 6 h after infarction. This release activated the myeloid differentiation primary response 88 (MyD88)—NF -κB pathway via the recognition of the TLR4/MD—2 complex, inducing the secretion of TNF -α and IL—1β by myocardial macrophages and triggering the NLRP3 inflammasome, which ultimately led to myocardial injury [85]. Furthermore, elevated serum levels of S100A8/A9 in patients with sepsis are significantly correlated with an increased risk of mortality [86–88]. In lung transplant recipients, the analysis of bronchoalveolar lavage fluid (BALF) through multiplexed immunoblotting demonstrated a tendency of elevated S100 protein family levels in patients with restrictive allograft syndrome (RAS) and bronchiolitis obliterans syndrome (BOS) when compared to those with stable graft function [89]. Nevertheless, distinct from the pro—inflammatory impact of S100A8/A9 in lung transplantation, within the context of renal transplantation, the S100A8/A9 heterodimer exhibited notably higher 5—year graft survival rates among patients with S100A8 ≥ 25 ng/mg and S100A9 ≥ 30 ng/mg compared to the low—expression group. Moreover, it was negatively correlated with the renal tubulointerstitial inflammation scores on renal biopsy one week after transplantation. Conversely, the levels of S100A8/A9 gradually declined in the renal tissues of patients suffering from chronic allograft nephropathy (CAN) [89].

Research on the immunomodulatory function of S100 proteins has indicated that S100A8, S100A9, and their heterodimeric calprotectin are capable of inducing immune tolerance through a multi—level mechanism. Firstly, calprotectin recruits G9a histone methyltransferase to the TNF -α promoter region via the RAGE receptor, leading to H3K9 demethylation and subsequently reducing TNF -α secretion in LPS—stimulated monocytes. This epigenetic silencing is a direct consequence of the decreased activity of the p38 MAPK pathway [90, 91]. Secondly, S100A8/A9 impaired T cell activation by inhibiting DCs MHC-II molecule expression and CD80/CD86 co-stimulatory molecules [92]. Meanwhile, it inhibited neutrophil ROS production and its chemotaxis mediated by adenosine metabolites [93, 94]. More crucially, the S100A8 homodimer and calprotectin initiated the NF -κB signaling pathway MDSCs through the RAGE. Moreover, the activation of MDSCs prompted the synthesis and secretion of the S100A8/A9 heterodimer, thereby giving rise to a vicious cycle encompassing the recruitment, activation, and secretion of calprotectin by MDSCs [94].

#### Histone

In 1884, Albrecht Kossel initially disclosed the presence of histones, which are highly conserved cationic nuclear proteins. These proteins can be classified into two primary subgroups, namely core histones and linker histones, according to their functions [95]. The core histones (H2A, H2B, H3, and H4) establish an octameric core via hydrophobic interactions. This core facilitates the initial compaction of DNA by coiling it into a left—handed helix, thereby forming nucleosomes, which are the fundamental structural units of chromatin [96, 97]. Conversely, the linker histones (H1 and H5) interact with internucleosomal DNA as well as the entry and exit sites of nucleosomes via their globular domains. This interaction modulates the accessibility of DNA to other nuclear proteins, consequently participating in fundamental genetic processes such as transcription, replication, and repair [98].

Neutrophil extracellular traps (NETs) represent fibrillar antimicrobial networks discharged by innate immune cells (predominantly activated neutrophils) through programmed cell death via NETosis. The formation of these structures is contingent upon the protein arginine deaminase 4 (PAD4)-mediated citrullination of histone H3. This process disrupts the electrostatic interactions between histones and DNA, thereby leading to chromatin decondensation and the generation of reticular structures with diameters ranging from 10 to 100 nm [99, 100]. NETs possess a core structure composed of a DNA backbone and antimicrobial proteins. Histones H2A/H2B account for approximately 70% of the total protein content within NETs and exert a crucial bactericidal function. Moreover, anti—H2A/H2B antibodies have been demonstrated to reduce the antimicrobial effectiveness of NETs [101, 102].

An increasing body of evidence suggests that the abnormal activation of NETs is correlated with the pathogenesis of sepsis, cancer, autoimmune disorders, and chronic obstructive pulmonary disease [103]. A substantial body of literature corroborates that histones are capable of mediating acute organ injury (AOI) through immune modulation via the activation of TLRs. As co—receptors of innate immunity, TLRs have been thoroughly documented to assume a crucial role in sterile inflammation mediated by extracellular histones functioning as DAMPs [104, 105]. Free histones (H2A/H3) initiate the activation of NF-κB/p38 MAPK via the TLR2/4-MyD88 pathway, thereby triggering a cascade of pro-inflammatory cytokines (e.g., IL-6, TNF-α), platelet aggregation, and activation of coagulation factor XII. Ultimately, this process induces disseminated intravascular coagulation (DIC) in sepsis. Notably, TLR2/4 double-knockout mice exhibit significantly improved survival rates after a lethal histone challenge. In contrast, treatment with an anti-TLR4 monoclonal antibody significantly reduces mortality in wild-type mice [102, 106]. Secondly, histone—DNA complexes induced a type I interferon storm and NLRP3 inflammasome activation via the TLR9—cGAS/STING pathway. This resulted in elevated IL—1βsecretion and an expansion of the necrotic area in hepatic ischemia/reperfusion injury, which was effectively inhibited by anti—H3/H4 antibodies [107, 108]. Furthermore, histones directly disrupt the cell membranes of endothelial or epithelial cells through cation—phospholipid interactions. This process results in the formation of transmembrane pores, which induce Ca^2+^ influx and cause the collapse of mitochondrial membrane potential. Ultimately, it leads to endothelial barrier dysfunction in cases of traumatic shock [109, 110]. Notably, endogenous C—reactive protein (CRP) impedes histone insertion and mitigates Ca^2+^ influx via competitive binding to cell membrane phospholipids, thereby enhancing the survival rate of septic patients with CRP levels ≥ 10 mg/L [111].

### Nucleic acid-based DAMPs

#### DNA

Immune cells, especially neutrophils, release chromatin as the primary source of extracellular DNA through NETosis [112]. The levels of extracellular DNA act as a specific biomarker for circulating NETs, and their detection in conjunction with NETs—associated proteins can accurately reflect the pathological state of certain diseases [113]. Apart from immune cells, necrotic vascular endothelial cells and parenchymal cells constitute the secondary principal source of extracellular DNA. Under hypoxic conditions or subsequent to uncontrolled injury, the majority of cells experience necrotic death, releasing intact genomic DNA through plasma membrane disruption [114].

Extracellular DNA contributes to immune activation and disease progression through diverse mechanisms. The concurrent elevation of DNA, histones, and HMGB1 levels has been shown to induce coagulation dysfunction in sepsis models [115, 116]. Earlier studies proposed that mammalian DNA has low immunogenicity. A groundbreaking study revealed that bacterial DNA containing unmethylated CpG motifs activates innate immunity via the TLR9—MyD88 pathway, while mammalian DNA exhibits immune tolerance due to methylation—mediated blockade of TLR9 binding [117–119]. Subsequent research demonstrated that the non—TLR pathway can surmount this limitation under specific circumstances. Through endocytic delivery by transfectants, cytoplasmic DNA sensors activate interferon regulatory factor 3 (IRF3) via either the cyclic guanosine monophosphate—adenosine monophosphate (cGAMP)—STING or receptor—interacting serine/threonine—protein kinase 1/3 (RIPK1/3) signaling cascades, ultimately leading to the production of IFN -β [120–122]. Both natural nucleosomal DNA and synthetic oligonucleotides conjugated to DNA autoantibodies form immune complexes that display enhanced activation efficiency through FcγRIIa receptor—mediated endocytosis compared to free DNA. These DNA—containing immune complexes demonstrate a greater capacity for activating plasmacytoid dendritic cells through FcγRIIa—mediated endocytic uptake than free DNA [123]. During this process, as DNA antigenic epitopes are exposed, the simultaneous activation of both TLR9 and cGAS initiates the TLR9—MyD88 signaling pathway, resulting in substantial production of IFN—α [123]. Protein enhancers such as LL37 bind to DNA through their C—terminal cationic domains to form LL37—DNA complexes. These complexes are endocytosed via low—density lipoprotein receptor—related protein 1 (LRP1) receptor—mediated endocytosis, thereby augmenting TLR9 activation and amplifying immune responses [124]. HMGB1 binds to the minor groove of DNA, stabilizing NETs structures, increasing the exposure of CpG motifs, and enhancing the activation of the dual TLR4/TLR9 pathway [115].

#### RNA

Extracellular ribonucleic acids (exRNAs) constitute a heterogeneous group of ribonucleic acids, including microRNAs (miRNAs), messenger RNAs (mRNAs), transfer RNAs (tRNAs), ribosomal RNAs (rRNAs), and long non—coding RNAs (lncRNAs). These molecules fulfill their extracellular functions either through autonomous actions or via molecular interactions. Each exRNA species makes a distinctive contribution to microenvironmental regulation, which is determined by its sequence specificity and modification pattern. Under physiological homeostasis, the concentrations of circulating exRNAs are maintained at baseline levels [125]. Nevertheless, in the context of pathological stimuli such as hypoxia, microbial infection, or tumorigenesis, the levels of exRNA demonstrate a dose—dependent increase, attaining concentrations 10—to 100—fold higher than the baseline [126].

ExRNA contributes to immune activation and disease progression via multiple mechanisms. In autoimmune disorders, such as psoriasis, the LL37—exRNA complex functions as a crucial pathogenic factor that mediates chronic inflammation through the activation of dual receptors. Specifically, LL37, which is the C—terminal fragment of the cathelicidin antimicrobial peptide, binds endogenous exRNA through protease—mediated processing to form stable complexes [127]. The complex undergoes endocytosis mediated by the RAGE—CD36 receptor axis, thus evading nuclease degradation. Subsequently, within endosomes, plasmacytoid dendritic cells (pDCs) recognize the released RNA ligands via TLR7, initiating the TLR7—MyD88 signaling cascade. This activation stimulates the secretion of IFN—α while facilitating dendritic cell maturation and Th1 immune polarization [128]. Myeloid dendritic cells (mDCs) activate the TLR8—TRAF6 pathway via TLR8 recognition, thereby inducing the release of IL—6/TNF—α and promoting neutrophil activation followed by NETosis. This cascade forms a self—perpetuating "injury—inflammation—injury" cycle in the pathogenesis of autoimmune diseases [129].

Experimental findings indicate that ribosomal exRNA exhibits a high—affinity binding to vascular endothelial growth factor (VEGF), resulting in the formation of VEGF—exRNA complexes. These complexes activate the VEGFR—2/neuropilin—1 signaling pathway. Such activation triggers intracellular calcium transients, which in turn disrupt the localization and phosphorylation of both tight junction and adherens junction proteins [130, 131]. Simultaneously, fluid shear stress induces endothelial cells to secrete exRNA, which competitively inhibits the binding of heparin—like glycosaminoglycan to VEGF, consequently enhancing VEGFR—2 phosphorylation [132]. This mechanism is manifested in stroke models as sagittal sinus thrombosis accompanied by vasogenic cerebral edema. The severity of this edema demonstrates a positive correlation with both the degradation of tight junction proteins and the swelling of astrocytic foot processes [133]. In chronic inflammatory conditions such as RA and atherosclerosis exRNA exhibits dose—dependent deposition in synovial tissues and atherosclerotic plaques, with concentrations significantly higher than those in healthy controls [134, 135]. In ischemia/reperfusion (I/R) injury, exRNA directly induces cardiomyocyte apoptosis via the TLR3/TRIF pathway while upregulating TNF-α expression. This TNF-α activation triggers NLRP3 inflammasome assembly through TNFR1, resulting in enhanced IL-1β/IL-18 secretion. Concurrently, exRNA mediates ROS generation via NADPH oxidase and activates MAPK signaling pathways, collectively exacerbating myocardial fibrosis [136, 137] exRNA functions as a cofactor for bacteria-host cell interactions and microbial invasion, with both components synergistically enhancing infection efficiency. Streptococcus pneumoniae binds exRNA through basic amino acid residues to form stabilized complexes, which subsequently activate fibrinogen to degrade the host extracellular matrix. This mechanism facilitates bacterial adhesion to and invasion of alveolar epithelial cells [127, 138].

### Mitochondria-associated DAMPs

Human mitochondria are subject to the dual genetic regulation of nuclear and mitochondrial DNA. As conventionally acknowledged, their core function is to supply energy to the cell via oxidative phosphorylation. Moreover, they participate in functions such as the preservation of calcium signaling homeostasis, the modulation of ROS levels, and the initiation of the immune response [139–141]. In the face of stressful conditions such as genetic mutations, viral infections, and aging, mitochondria can modulate their own function and cellular metabolism through mitochondrial fission dynamics [142] and fusion [141, 143]. Nevertheless, upon encountering extreme stress, mitochondria and their constituents can be either actively secreted or passively discharged into the extracellular milieu, thereby participating in the preservation of physiological homeostasis and the initiation of pathological inflammatory responses [144]. Recently, it has been found that mitochondria are released in various forms, including intact mitochondria, mitochondrial fragments, vesicle-encapsulated components (exosome-encapsulated mtDNA), and free molecules (circulating mtDNA) [145].

In contrast to circulating nuclear DNA (cirr—nDNA), which is stabilized by nucleosomes, naked cyclic mtDNA, the most extensively studied mitochondrial—derived component, necessitates binding to granular structures to withstand degradation [146, 147]. Chiu et al. utilized differential centrifugation to substantiate that cirr—mtDNA exists in multiple physical states. Specifically, 75.7% is bound to intact mitochondria or large extracellular vesicles (EVs), 18.4% is associated with small EVs, and 5.9% forms complexes with exosomes or protein structures. This finding suggests that its release encompasses both active and passive mechanisms [148]. Recent studies have revealed that EVs, particularly exosomes, can transport mitochondria-derived components, and that their mtDNA exists in multiple forms [149–152]: Sansone et al. first identified intact mitochondrial genes within exosomes derived from breast cancer cells [153]; in plasma exosomes of healthy individuals, mtDNA primarily exists in fragmented forms; these exosomes can also package mtDNA-derived mRNAs [154]. In recent times, it has been discovered that autophagy—deficient cells possess the ability to bind mitochondria—derived mRNAs, which are crucial constituents of mitochondria—derived mRNAs. Moreover, it has been found that autophagy—deficient cells exhibit a notably higher abundance of intact mtDNA in exosomes. This phenomenon may be attributed to the secretion of mtDNA through the MVB—exosome pathway as a result of the delayed removal of damaged mitochondria [155].

Since 2004, when Zhang et al. first demonstrated that endogenous mtDNA induces the secretion of the pro-inflammatory cytokine TNF-α via the NF-κB pathway [156, 157]. Currently, it is acknowledged that mtDNA functions as a DAMP to trigger multiple PRR signaling pathways that mediate the inflammatory response. TLR9, serving as the initial extracellular DNA sensor, specifically identifies unmethylated CpG motifs in bacterial or viral DNA within the endolysosomal compartment. TLR9 can also detect analogous sequences in mitochondrial DNA and activates a signaling cascade involving nuclear factor NF -κB and interferon regulatory factor 7 (IRF7) via the MyD88 adaptor protein, thereby inducing the secretion of inflammatory factors such IL—6 and IFN -α [158]. Studies have shown that this pathway is widely activated in non-alcoholic steatohepatitis [159] and sepsis [160]. In type 2 diabetes [161] and sickle cell disease [162], The NLRP3 inflammasome has been reported to drive chronic low-grade inflammation by recognizing cytoplasmic mtDNA or mitochondrial ROS, recruiting the ASC adaptor protein and pro-caspase-1, and mediating IL-1β/IL-18 maturation and cell death. In contrast, caspase-1-mediated GSDMD cleavage can trigger tissue necrosis cascades in ischemia–reperfusion injury [163]. Furthermore, Torralba et al. have indicated that cytoplasmic mtDNA recognized by cGAS stimulates the secretion of IFN—β and C—X—C motif chemokine ligand 10 (CXCL10). This signaling pathway is especially notable in T cell—derived EVs. In this context, activated T cells discharge mtDNA—histone complexes through EVs, thereby initiating an antiviral—like reaction in non—immune cells [164].

Apart from mtDNA, mitochondrial double-stranded RNA (mt-dsRNA), mitochondrial proteins, lipids, and metabolites can also function as DAMPs to initiate inflammatory responses via specific receptors. The mitochondrial genome is transcribed via multiple cis—elements, leading to the production of mt—dsRNA [165]. When oxidative stress (e.g., H_2_O_2_treatment) leads to an increase in mitochondrial membrane permeability, mt-dsRNA leaks into the cytoplasm through voltage-dependent anion channels (VDAC) to form a pool of stress granule-associated free dsRNA [166]. In a melanoma research, mt-dsRNA was shown to mediate distinct immune responses via two members of the retinoic acid-inducible gene-I (RIG-I)-like receptor (RLR) family. Melanoma differentiation-associated protein 5 (MDA5) binds to long-stranded mt-dsRNA, recruits mitochondrial antiviral-signaling (MAVS) proteins to mitochondria-associated endoplasmic reticulum membranes (MAMs), activates the NF-κB pathway, and induces the expression IFN-β, IL-15, and IFN-λ1. This results in the secretion of IFN-β and IL-6 [167]. Meanwhile, RIG-I recognizes 5'-triphosphorylated mt-dsRNA fragments, interacts with MAVS through its caspase activation and recruitment domain (CARD), and localizes to mitochondria. This interaction triggers IRF7-mediated IFN-α/β production while simultaneously initiating autophagic degradation via ULK1 ubiquitylation [168].

Mitochondrial transcription factor A (TFAM), a mtDNA-binding protein that demonstrates 32% sequence homology with HMGB1, activates dendritic cells through the TLR4-MyD88 pathway. Under conditions of oxidative stress, TFAM mediates the synergistic activation of both the cGAS-STING pathway and NLRP3 inflammasomes in response to mtDNA [169].

Cardiolipin (CL), an unsaturated tetra—acylated phospholipid primarily situated in the inner mitochondrial membrane, undergoes translocation to the outer mitochondrial membrane upon oxidation. Oxidized CL activates the NF -κB via the TLR4-MD-2 complex. Meanwhile, its biconical structure directly binds to the NOD—like receptor family, NLRP3 to promote inflammasome assembly [170]. Notably, anti—cardiolipin (anti—CL) autoantibodies trigger the formation of NETs via Fcγ receptor IIa (FcγRIIa)—dependent mechanisms, consequently facilitating a thrombotic—inflammatory cascade in antiphospholipid syndrome (APS) [171].

**Fig. 4:**
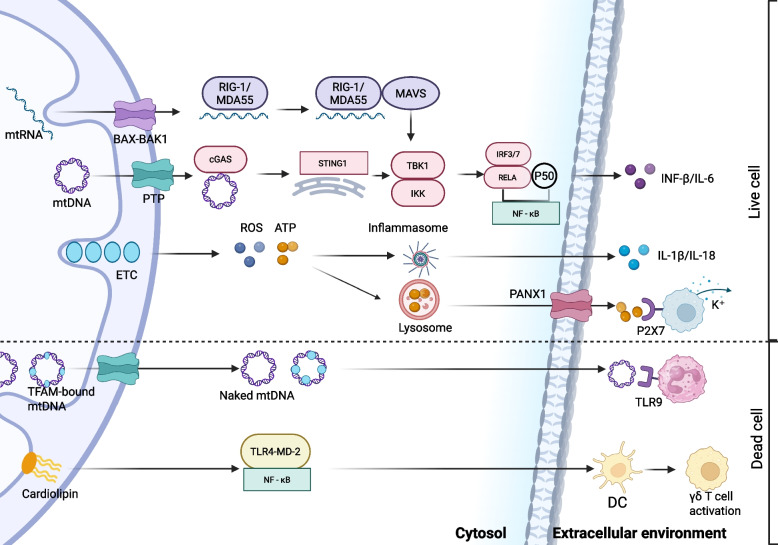
Mitochondria-derived DAMPs. Mitochondrial RNA (mtRNA) is released through the BAX-BAK1 channel, activating RIG—1/MDA5 and subsequently signalling via MAVS. Mitochondrial DNA (mtDNA) is released via PTP, recognised by cGAS and activates STING1, which in turn acts on TBK1 and IKK, ultimately affecting IRF3/7 and NF-κB, and contributing to INF-β/IL-6 production. ROS and ATP produced by the mitochondrial electron transport chain (ETC) act on inflammatory vesicles to promote IL-1β/IL-18 release. In addition, lysosome-associated PANX1 and P2X7 are involved in K^+^ efflux and apoptosis. After cell death, TFAM-bound mtDNA and naked mtDNA activate TLR9 to mediate the inflammatory response, and cardiolipin activates TLR4-MD-2 and NF-κB, ultimately triggering dendritic cell (DC) and γδ T cell activation

Among DAMPs, ATP as a central product of mitochondrial oxidative phosphorylation, is synthesized at the inner mitochondrial membrane through ATP synthase-catalyzed conversion of ADP and inorganic phosphate (Pi), playing a pivotal role in maintaining intracellular energy homeostasis. During mitochondrial stress or immune cell activation, ATP undergoes extracellular release via either SNARE protein complex-dependent vesicular exocytosis or pannexin-1 channel-mediated diffusion, thereby acquiring DAMP functionality [172]. Studies demonstrate that extracellular ATP activates the TLR4 pathway to upregulate NLRP3 and pro-IL-1β transcription. The ATP-P2X7 receptor interaction subsequently mediates potassium efflux, inducing conformational changes in the NLRP3 NACHT domain. This promotes ASC oligomerization and pro-caspase-1 recruitment to assemble functional inflammasomes, ultimately leading to GSDMD cleavage and pyroptotic cell death [173]. Furthermore, extracellular ATP has been demonstrated to interact with circulating mannose-binding lectin (MBL), thereby initiating complement activation and serving as a potent inflammatory stimulus [174]. These non-nucleic acid DAMPs function synergistically with mtDNA to establish a multi-tiered inflammatory activation network, which plays pivotal roles in both infectious and autoimmune pathologies (Fig. 4).

## Pattern Recognition Receptors (PRRs)

Serving as the core sensors of the innate immune system, PRRs initiate multi—level immune defenses through the recognition of PAMPs and DAMPs. Among them, TLRs, which are among the earliest identified PRRs in the innate immune system, play a crucial role in mediating inflammatory responses [175, 176]. Since the discovery of TLR4, numerous PRRs and their corresponding ligands have been identified. Based on protein domain homology, PRRs can be systematically classified into five major types: TLRs, retinoic acid-inducible gene I (RIG-I)-like receptors (RLRs), nucleotide-binding oligomerization domain (NOD)-like receptors (NLRs), absent in melanoma 2 (AIM2)-like receptors (ALRs), and C-type lectin receptors (CLRs). RAGE although functionally a PRR, exhibits distinct structural and functional characteristics that preclude its classification into the traditional five families and instead places it within the immunoglobulin superfamily. Upon recognition of their ligands, whether PAMPs or DAMPs, PRRs initiate a cascade of signaling events, activating complex downstream pathways and eliciting diverse biological responses. In this section, we focus on the PRRs involved in DAMPs recognition: TLRs, NLRs, CLRs, and RAGE.

### Toll-like receptors (TLRs)

TLRs are type I transmembrane glycoproteins characterized by a wide—ranging cellular distribution. In the immune system, the expression of TLRs has been detected in macrophages, DCs, B lymphocytes, natural killer (NK) cells, and specific subsets of T lymphocytes. Additionally, these receptors are also present on epithelial cells, endothelial cells, and fibroblasts. Based on their subcellular localization, TLRs can be classified into two distinct categories: (1) cell—surface TLRs (including TLR1, TLR2, TLR4, TLR5, and TLR6) and (2) intracellular TLRs (such as TLR3, TLR7, TLR8, and TLR9) that are located in endosomal compartments, where they recognize and bind to their corresponding ligands [176, 177]. All TLRs possess a conserved tripartite architecture, which consists of an extracellular domain, a transmembrane domain, and an intracellular domain. The extracellular domain encompasses leucine-rich repeats (LRRs), which play a role in mediating specific ligand recognition and subsequent pattern recognition. The intracellular domain is characterized by a Toll/interleukin-1 receptor (TIR) homology domain, which is crucial for signal transduction, allowing TLRs to activate downstream signaling cascades and initiate immune responses [178]. In mammalian cells, TLRs are biosynthesized within the endoplasmic reticulum (ER) and then transported to their corresponding cellular sites. This trafficking mechanism necessitates the support of endoplasmic reticulum—resident chaperone proteins, such as the protein associated with TLR4 A (PRAT4A) and Unc93B1 [179–181]. The deficiency of molecular chaperones gp96 and PRAT4A disrupts the ligand recognition capability of the majority of TLRs (excluding TLR3), thereby compromising the host's capacity to initiate effective immune responses. This molecular aberration results in a significantly reduced production of pro—inflammatory cytokines, type I interferons, and chemokines subsequent to pathogenic challenge [182, 183].

Current experimental evidence indicates that DAMPs, similar to PAMPs, require distinct groups of co-receptors and accessory molecules for their recognition by TLRs. Mechanistically, DAMPs can be comprehensively classified into two primary categories: endogenous alarmins, such as HSP60, HSP70, and S100 proteins, which interact with specific TLRs through well—defined molecular interactions via CD14 and/or MD—2. The second category of DAMPs utilizes alternative co-receptor systems that are molecularly different from the CD14/MD—2 complex [184–186]. For instance, in patients with systemic lupus erythematosus (SLE), autoantibodies directed against double—stranded DNA (dsDNA) and nucleosomes can trigger the activation of DCs via TLR2 when complexed HMGB1. Analogously, HMGB1—DNA immunocomplexes facilitate the activation of pDCs and B cells through the TLR9 signaling pathway, a process in which the RAGE, a member of the immunoglobulin superfamily, is involved [187].

### Nucleotide-binding oligomerized structural domain-like receptors (NLRs)

NLRs constitute another principal category of pattern recognition receptors that have undergone comprehensive exploration subsequent to the discoveries of TLRs. These cytoplasmic receptors demonstrate extensive phylogenetic conservation, being present in both invertebrate and vertebrate taxa. In Homo sapiens, the NLR family encompasses 22 well-characterized members, all of which are solely localized within the cytoplasmic compartment [188, 189]. All members of the NLR family possess three conserved structural domains that determine their functional mechanisms: (1) The N—terminal effector domain (e.g., CARD, PYD, or BIR domains) mediates interactions with adaptor proteins and downstream signaling effectors, acting as the primary transducer of receptor activation signals; (2) The central NACHT domain (nucleotide—binding and oligomerization domain) promotes ATP—dependent auto—oligomerization, serving as the molecular switch for NLR activation; (3) The C—terminal leucine—rich repeat (LRR) region shows variable lengths among family members and functions as the ligand—sensing module for detecting pathogenic or danger signals [190, 191].

As crucial recognition elements of the innate immune system, NLRs possess extensive recognition capacities. They can not only precisely identify diverse PAMPs but also recognize DAMPs, such as extracellular ATP, hyaluronic acid degradation fragments, sodium urate (MSU) crystals, uric acid, and cholesterol crystals [192]. Activated NLRs possess the capacity to execute a diverse array of pro-inflammatory and signal transduction functions, which play a role in the progression of diseases. For instance, NLRP3 identifies DAMPs such as ATP and triggers the assembly of inflammasomes, the core of which is composed of NLRs, ASC and pro-CASP1 [193]. Activated inflammatory vesicles facilitate the maturation of pro—IL—1β/18 through caspase—1. This process potentiates the inflammatory response and triggers focal death, leading to the release of a greater quantity of DAMPs. Consequently, a positive feedback loop is established, which amplifies the inflammatory process [194, 195]. Research has indicated that within lung—associated fibrosis, the recognition of ATP and uric acid by the NLRP3 initiates the recruitment of IL—1 and tissue inhibitor of metalloproteinases 1 (TIMP1) in inflammatory cells. This process intensifies the inflammatory response on the basis of the pre—existing inflammation and facilitates extracellular collagen synthesis, ultimately contributing to the progression of pulmonary fibrosis [196]. In liver disease, S100A9 is released during liver necrosis, which binds to NLRP3 on hematopoietic stem cells and myofibroblasts, mediating an increase in the expression of TNF and IL-17 [197], which triggers an inflammatory response and promotes the synthesis of extracellular collagen, ultimately leading to liver fibrosis [198]. In addition, activation of Smad2/3 by NLRP3 phosphorylates vascular endothelial cells and transforms them into myofibroblasts, thereby increasing the production of α-SMA and MMP9 [199].

### C-type lectin receptors (CLRs)

CLRs serving as phagocytic PRRs, exhibit a differentiating mechanism compared to signaling-activated PRRs. They assume a crucial position in immune defense by identifying and binding PAMPs or DAMPs via their PRR domains. Subsequent to binding, these complexes are internalized into cytoplasmic vesicles, wherein pathogens are directly digested and eradicated, thus exerting control over infections [200, 201]. CLRs identify both self and non-self carbohydrate structures via their carbohydrate recognition domains (CRDs), a process that necessitates the presence of Ca^2+^. These receptors are extensively expressed on immune cells, including macrophages and DCs, as well as in specific tissues [202–205]. Based on their cellular localization,CLRs can be classified into two main types: transmembrane receptors and secretory receptors [203, 206, 207]. The primary representatives of secreted pattern recognition receptors are the collectin family, a subset of extracellular pattern recognition molecules [208]. Transmembrane receptors can be subdivided into type I and type II [209]. Type I receptors are characterized by an extracellular N-terminus and multiple CRDs. In contrast, type II receptors exhibit an intracellular N-terminus and only one CRD [210, 211]. Research has demonstrated that most CLRs function as membrane-bound receptors actively participating in antigen presentation processes [202].

CLRs can be classified into four categories according to their cytoplasmic signaling motifs. Among them, macrophage-induced type C lectin (MINCLE), which is closely related to DAMPs, recognizes SAP130, cholesterol sulfate, and cholesterol crystals, etc. [212, 213], and upon activation, induces the production of cytokines such as TNF-α, IL-6, and chemokines [214, 215]. It has been shown that MINCLE senses tissue damage by recognizing SAP130 released from necrotic cells, induces secretion of inflammatory factors and promotes neutrophil infiltration [216]. In idiopathic pulmonary fibrosis, binding of SAP130 to MINCLE increases interstitial inflammatory factor secretion and promotes fibrosis formation [217]. In hepatic tissues, MINCLE is predominantly detected in Kupffer cells and macrophages. When DAMPs interact with MINCLE on the cell membrane, they trigger the cellular secretion of inflammatory factors and facilitate the infiltration of M1 macrophages [218]. DAMPs released from dead adipocytes in obesity activate macrophage MINCLE, which induces myofibroblast production and promotes fibrotic gene expression [219]. Inhibition of MINCLE-mediated inflammatory response alleviates renal fibrosis [220]. In addition, CTLRs are involved in the regulation of interstitial lung disease by regulating M-CSF expression [221].

### Receptor for Advanced Glycosylation End products (RAGE)

RAGE is a transmembrane glycoprotein with a molecular weight ranging from 50 to 55 kDa, which belongs to the immunoglobulin superfamily. In terms of structure, RAGE is composed of three domains: an extracellular ligand—binding region, a hydrophobic transmembrane domain, and a cytoplasmic tail. RAGE engages in interactions with a variety of DAMPs, such as HMGB1, S100 proteins, histones, amyloid β, and extracellular DNA, thus mediating cellular responses to these ligands [222]. The cytoplasmic domain of RAGE demonstrates a high degree of sequence conservation among primates and rodents. This domain is characterized by a highly acidic region that facilitates interactions with multiple signaling molecules, which is a crucial prerequisite for ligand—dependent signal transduction. Truncation of this domain perturbs downstream signaling pathways and alleviates disease pathology [223]. The transmembrane helices of RAGE contain highly conserved motifs that not only promote helix-helix homodimerization but may also play a role in signal transduction [224]. The extracellular region of RAGE comprises a variable (V) immunoglobulin (Ig) domain followed by two constant Ig domains (C1 and C2), which are connected by a flexible linker [222]. The V-C1 domain features a hydrophobic cavity on its surface and is enriched with positively charged arginine (Arg) and lysine (Lys) residuess [225]. In contrast, the C2 domain is composed primarily of acidic amino acids, resulting in an overall negatively charged surface [226]. Due to the predominantly negatively charged regions in its ligands, RAGE exhibits binding affinity for the positively charged V-C1 domain [227]. In its monomeric form, RAGE displays merely a feeble binding affinity towards various ligands, which implies that receptor multimerization is indispensable for efficient ligand binding. Research has indicated that heparan sulfate plays a crucial role in stabilizing RAGE oligomerization, encompassing V—domain—mediated ionic self—association in dimers and the generation of hexameric complexes [228].

RAGE is ubiquitously expressed among diverse cell types, encompassing vascular smooth muscle cells, endothelial cells, neoplastic cells, monocyte—derived macrophages, and adipocytes. Significantly, the expression of RAGE is markedly upregulated under various pathological circumstances, especially in atherosclerosis, diabetes mellitus, CVD, RA, Alzheimer's disease (AD), and immune—related disorders [229–233]. Emerging evidence indicates that RAGE plays a significant role in the pathogenesis and progression of various malignancies [234]. In colorectal cancer, the HMGB1-RAGE axis promotes autophagy via ERK-mediated phosphorylation of Drp1, a mechanism contributing to both chemoresistance and tumor cell regeneratio [235]. In AD, RAGE-mediated binding to amyloid-β (Aβ40/42) induces neurotoxic aggregate formation and promotes disease progression by enabling the blood–brain barrier (BBB) penetration of Aβ, while simultaneously upregulating pro-inflammatory cytokines and endothelin-1 [236]. Moreover, HMGB1 facilitates the RAGE—Mac—1 interaction between endothelial cells and leukocytes in a dose—dependent fashion, simultaneously activating the NF—κB pathway in neutrophils [237, 238] (Table 1).

**Table 1:** Classification of DAMPs, receptors, and associated diseases

DAMPs		Acceptor	Illnesses
Protein type	HMGB1	TLR2, TLR4, RAGE, TIM3	Tumors: Hematopoietic malignancies [239]; Breast cancer [240]; Colorectal cancer [241]; Melanoma of the skin [242] ...; Myocardial infarction [243]; Atherosclerosis [244]; Cardiometabolic disorders [245]; Inflammation of the lungs [246], Idiopathic pulmonary fibrosis [247]; Acute lung injury [248]; Liver failure [249]; Alcoholic liver disease [250]; Acute kidney injury [251]; Scheugelen's Syndrome [252]; Periodontitis [253]; Septica [254]; Neurodegenerative pathologies [255]; Systemic lupus erythematosus [256]
	HSP	TLR2, TLR4, RAGE, CD14	Tumors [257] : Breast, Colorectal Cancer; Liver Failure [258]; Atherosclerosis; Diabetes; Lupus; Multiple Sclerosis; Rheumatoid Arthritis; Sepsis [259]
	S100 protein	TLR2, TLR4, RAGE.	Tumors [260, 261]: lung cancer, colorectal cancer, leukemia; cardiovascular inflammation [262]; liver failure [258]; idiopathic pulmonary fibrosis [247]; rheumatoid joints; sepsis; systemic sclerosis [259]; renal injury, renal fibrosis [263]
	histone	TLR2, TLR4, NLRP3, NOD2	Pneumonia, respiratory distress syndrome [264]; Liver inflammation [106]; Liver failure [258]
Ribonucleic acid (RNA or DNA)	DNA	cGAS-STING, AIM2, TLR9	Cardiovascular Disease [265]; Heart Failure [266]; Non-Alcoholic Fatty Liver Disease [267]; Fatty Liver [268]; Lupus [269]
	RNA	TLR2, TLR4	Atherosclerosis [244]; Acute Lung Injury; Idiopathic Pulmonary Fibrosis [270]; Acute Liver Injury [271]; Fatty Liver Disease [268]
Mitochondria-associated DAMPs	mtDNA, formyl peptide, cardiolipin	cGAS-STING	Cardiovascular Diseases [265, 272]; Respiratory Diseases [273]; Rheumatoid Arthritis [274]; Intestinal Ischemia Reperfusion Injury [275]; Neuroinflammatory and Neurodegenerative Diseases [276]; Non-Alcoholic Fatty Liver Disease [267]; Liver Failure [258]; Alcoholic Liver Disease [277]; Urinary Tract Obstruction [278];Acute Kidney Injury [279]
Other	IL-1α, IL-33	ST2	Tumors [280, 281]; Liver failure [258]; Urinary tract obstruction [278]
	ATP, ADP	P2X7-NADPH ; NLRP3	Nonalcoholic Fatty Liver Disease [267]; Atherosclerosis [282]; Fatty Liver Disease [268]
	Uric acid	NLRP3	Non-alcoholic fatty liver disease [267]; Renal fibrosis, nephropathy [283]; Urinary tract obstruction [278]
	oxLDL	TLR4	Cardiovascular Diseases [284]; Autoimmune Diseases [285]

## The DAMP signaling transduction mechanism based on PRR classification

When DAMPs bind to PRRs, they initiate multiple downstream signaling cascades, resulting in inflammatory responses, immune modulation, or cell death. The signaling pathways activated by different PRRs (e.g., TLRs, NLRs, RLRs, CLRs, cGAS-STING, etc.) differ and can be classified into the following categories based on the receptor type.

### The TLRs-NF-κB/MAPK pathway: a central mechanism of DAMPs-induced inflammatory response

**Fig. 5:**
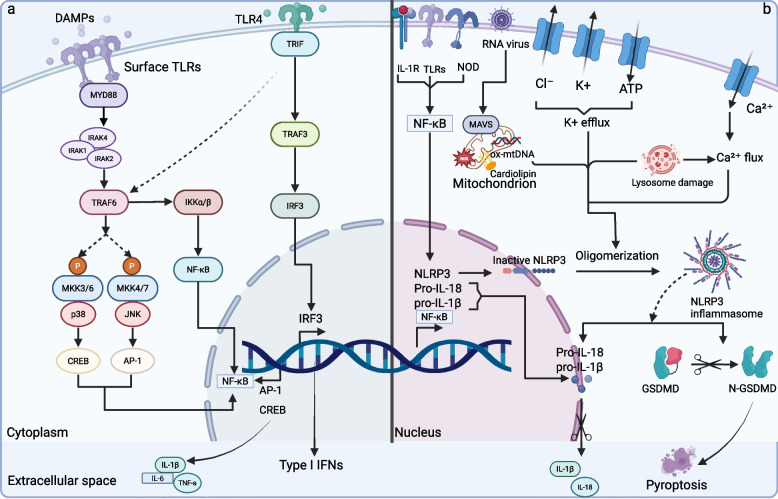
DAMPs and immune signal transduction. The left a figure demonstrates that TLRs bind to ligands to form homo-/heterodimers, and after dimerization, their TIR domains recruit the adaptor protein TIRAP, which binds to the junction protein MyD88 and catalyzes the kinase of IPAKs, activates TRAF6, which activates the IKK complex and activates NF-κB, which enters the nucleus and induces the up-regulation of transcription, and promotes the transcription of pro-inflammatory cytokine (e.g., TNF-α, IL-1β, IL-6) transcription. Meanwhile, TRAF6 also catalyzes MKKs, activates the JNK/p38 pathway, and induces AP-1 transcription, which in turn mediates RNA regulation.TLR3 and TLR4 activate the TRAF3-IRF3 axis through the TRIF-dependent pathway to induce type I interferon production. The right b panel shows the classical activation pathway of NLRP3 inflammasome: after sensing signals from mitochondrial ROS, ionic currents (K^+^ efflux, Ca^2+^ efflux), and lysosomal damage by the intracellular NLRP3 receptor, it assembles with ASC proteins to form an inflammasome that cleaves and activates caspase −1. Caspase −1, on the one hand, cleaves pro—IL—1β, on the other. Caspase-1, on the one hand, cleaves pro-IL-1β and pro-IL-18 as mature cytokines that mediate the inflammatory cascade; on the other hand, it cleaves GSDMD proteins to form N-GSDMD, which drives cellular pyroptosis and releases inflammatory contents to amplify the immune response

TLR binds to ligands to form homodimers or heterodimers. Following dimerization, their Toll-IL1R (TIR) domains recruit adaptor proteins. Based on the specific adaptor proteins involved (including MyD88, TRIF, TIRAP/MAL, or TRAM), the TLR signaling pathway is divided into two primary branches: the MyD88-dependent and the TRIF-dependent pathways. These pathways operate both independently and in an interconnected manner, jointly determining the intensity of the host inflammatory response and balancing the immune response by regulating key transcription factors such as NF-κB, MAPK, and IRF3 [286]. When TLRs signal through MyD88, the MyD88-dependent pathway leads to the activation of multiple signaling cascades, including the MAPK pathway and the NF-κB pathway. Activation of NF-κB induces the expression of pro-inflammatory cytokines like TNF-α, IL-1β, and IL-6. Concurrently, activation of MAPK (p38/JNK/ERK) activates AP-1, enhances mRNA stability, and promotes immune cell infiltration, all of which collectively orchestrate the inflammatory response [286–288]. The MyD88-dependent pathway is common to all TLRs. In contrast, TLR3 and TLR4 utilize the adaptor protein TRIF to activate TRAF3 and IRF3, which then induce type I interferon production (Fig. 5) [288]. The DAMPs-TLRs signaling pathway has been implicated in a variety of disorders, including neoplasms, cardiac diseases, lung inflammation, and autoimmune diseases [289, 290]. For instance, mtDNA released into circulation after acute myocardial infarction may exacerbate ischemia–reperfusion injury via the TLR9-p38 MAPK pathway, thereby adversely affecting the myocardium [163]. MyD88 exhibits both protective and pathogenic roles in most forms of cardiovascular disease. For instance, MyD88 in innate cells can lead to atherosclerosis, while MyD88 in adaptive cells confers protection [288]. Similarly, HMGB1-TLR-dependent pro-inflammatory mechanisms play a crucial role in the pathogenesis of autoimmune diseases [291]. In SLE, the HMGB1-TLR4 interaction promotes neutrophil NETosis, leading to the release of more HMGB1 and nuclear DNA, forming a positive feedback loop [256].

### NLRs-Inflammasome Pathway: DAMP-mediated NLRP3 Inflammasome Activation, Pyroptosis, and IL-1β Release

Activated NLRs manifest diverse functionalities, which can be comprehensively classified into four primary functions: inflammasome formation, signal transduction, transcriptional activation, and autophagy. This review specifically centers on the inflammasome activation mediated by NLRs. Among them, NLRP3 inflammasomes are the most intensively investigated, commonly present in neutrophils, monocytes, dendritic cells, macrophages, and non-hematopoietic cells. The canonical architecture of the NLRP3 inflammasome consists of NLRP3, ASC, and pro-caspase-1. The assembly of the NLRP3 inflammasome is a sophisticated process contingent upon dual-signal activation, encompassing two pivotal steps: priming and activation. The priming phase is initiated by DAMPs via pattern recognition receptors such as TLRs, activating the NF-κB signaling pathway and upregulating the expression of genes such as NLRP3, pro-IL-1β, and pro-IL-18. This phase also depends on caspase-8 and the FADD complex, facilitating the nuclear translocation of NF-κB, and involves post-translational modifications of NLRP3 (e.g., the deubiquitinase BRCA1/BRCA2-containing complex subunit 3 (BRCC3) removing the ubiquitin chain to stabilize NLRP3, or phosphorylation mediated by Syk/Jnk. The activation phase necessitates various intracellular stimuli (e.g., K^+^ efflux, Ca^2+^ mobilization, mitochondrial ROS, or lysosomal rupture) to trigger NLRP3 oligomerization. Activated NLRP3 recruits the adaptor protein ASC through its pyrin domain (PYD), which subsequently recruits pro-caspase-1 through its CARD, forming a functional inflammasome complex. Ultimately, caspase-1 cleaves pro-IL-1β and pro-IL-18 to generate and secrete their active forms, thereby initiating an inflammatory response. Lysosomal damage and the release of cathepsins can also partially promote NLRP3 activation [284]. Caspase-1 also cleaves GSDMD, which subsequently translocates to cell membranes to form pores, thereby mediating the release of pro-inflammatory cytokines and inducing cellular pyroptosis. This form of pyroptosis leads to the release of DAMPs and an exacerbated inflammatory response, creating a vicious cycle (Fig. 5) [191, 286]. In addition to the classical pathway described, a non-classical pathway (caspase-4/5/11-dependent) for NLRP3 inflammasome activation also exists and is involved in the response to DAMPs [272]. Furthermore, findings from a mouse model of unilateral ureteral obstruction suggest that NLRP3 promotes TGF-β signaling, R-Smad activation, and epithelial-mesenchymal transition in renal tubular epithelial cells through a mechanism independent of its classical inflammasome function [199]. The non-canonical pathway (dependent on caspase-4/5/11) directly cleaves GSDMD to form membrane pores upon recognition of intracellular LPS, inducing K^+^ efflux and indirectly activating NLRP3. Additionally, caspase-11 can promote inflammation by activating pannexin-1 channels to release ATP, further amplifying inflammatory signals. Both mechanisms ultimately lead to the activation of caspase-1 and the maturation and secretion of IL-1β and IL-18; however, DAMPs tend to be more associated with organelle dysfunction triggers, whereas the non-canonical pathway is closely related to pathogen recognition [284].

### CLR-mediated signaling pathway: DAMP-induced immune recognition, activation, and suppression

**Fig. 6:**
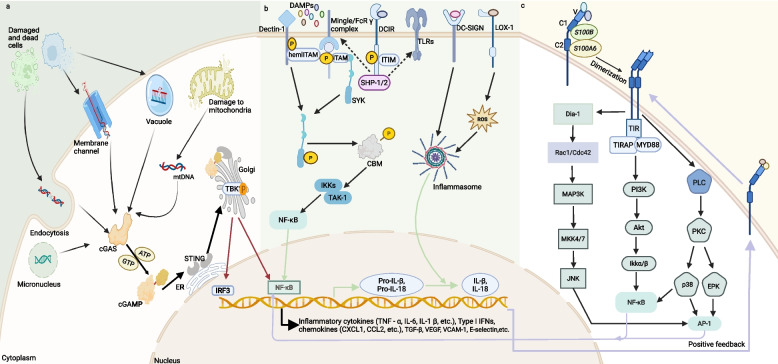
DAMPs-mediated proinflammatory signal transduction. This schematic illustrates the activation mechanisms of three innate immune signaling pathways: **a** In the cGAS-STING pathway, cytosolic dsDNA activates cGAS to produce cGAMP, which induces STING translocation and subsequently activates the IRF3/NF-κB pathway via TBK1, leading to the production of pro-inflammatory cytokines; **b** In the DAMP-sensing pathway, activating receptors (e.g., Dectin-1) engage the SYK/CBM complex to trigger the TAK1-IKKs-NF-κB cascade, promoting pro-IL-1β transcription, while inhibitory receptors (e.g., DCIR) suppress SYK activity through SHP-1/2, and the NLRP3 inflammasome cleaves pro-IL-1β to generate mature IL-1β; **c** In the RAGE signaling pathway, ligand-bound RAGE activates NF-κB via PI3K-AKT-IKK or AP-1 via Rac1-MAP3K-JNK, thereby releasing cytokines/MMPs, while HMGB1-RAGE amplifies NF-κB/AP-1 signaling through the PLC-PKC axis, establishing a pro-inflammatory feedback loop

CLRs play a diverse role in immune responses. When bound to their specific ligands, different CLRs can differentially influence cellular processes such as endocytosis and phagocytosis, as well as modulate pro-inflammatory or anti-inflammatory outcomes, a function primarily determined by their generalized signaling systems. Furthermore, CLRs bridge innate and adaptive immunity through their involvement in antigen internalization, antigen presentation, and T-cell activation. Based on their intracellular signaling motifs, CLRs are broadly classified into four major categories: 1) ITAM-coupled CLRs (e.g., Mincle/FcRγ complex): Mincle forms a complex with the Fc receptor γ chain. This complex then recruits spleen tyrosine kinase (SYK) via the immunoreceptor tyrosine activation motif (ITAM). Subsequently, SYK activation leads to the activation of the NF-κB signaling pathway, which induces the expression of pro-inflammatory cytokines and chemokines. Moreover, this pathway promotes the polarization of macrophages towards an M1-type pro-inflammatory phenotype, thereby playing a crucial role in anti-infective immunity and inflammatory responses. 2) hemITAM types (e.g., Dectin-1): These CLRs activate NF-κB through the Syk-CARD9/Bcl10/MALT1 pathway, consequently influencing the overall immune response. 3) ITIM types (e.g., DCIR): CLRs with immunoreceptor tyrosine-based inhibitory motifs (ITIMs) negatively regulate signaling by other PRRs through the recruitment of SHP-1/2 phosphatases. 4) CLRs lacking typical ITAM or ITIM signaling motifs (e.g., DC-SIGN and LOX-1): The diverse regulatory functions of these CLRs are dependent on their interactions with other scaffolding or signaling molecules (Fig. 6) [213, 218].

### RAGE pathway: DAMP-induced altered cellular function, inflammatory cascade, and differentiated signaling responses

Activation of the RAGE triggers multiple intracellular signaling pathways, including mitogen-activated protein (MAP) kinases (ERK 1/2, p38, SAPK/JNK), STAT3, Akt, and Rho GTPases (Rac1, Cdc42). These, in turn, activate downstream transcription factors, including NF-κB, EGR-1, and SP-1. These pathways lead to the expression of a range of pro-inflammatory genes, including VCAM-1, IL-6, TNF-α, and other immune modulators [227]. RAGE activates intracellular signaling by preassembling into dimers and multimers on the cell surface. Upon binding to ligands, multimeric RAGE undergoes conformational changes in its intracellular structural domains, enabling interactions with its intracellular chaperones. This process involves the activation of Rho GTPases and kinases that subsequently activate transcription factors. These signaling pathways lead to alterations in gene expression and cellular functions, including migration, survival, inflammation, and the upregulation of RAGE expression itself [222, 227]. Different DAMPs trigger distinct RAGE-dependent signaling pathways. For example, HMGB1 enhances the immunosuppressive function of regulatory T cells (Tregs) via a RAGE-mediated mechanism and limits the number and activity of other T cells, which may influence immune responsiveness in chronic inflammatory conditions [56]. Additionally, S100B and S100A6 interact with different extracellular structural domains of RAGE. At equal concentrations, S100B promotes cell proliferation, whereas S100A6 induces apoptosis. Furthermore, binding of these S100 proteins to RAGE induces reactive oxygen species formation. Specifically, S100B-RAGE interaction primarily activates the PI3K/AKT pathway and NF-κB transcription factors, while S100A6-RAGE engagement triggers the activation and phosphorylation of JNK, leading to different downstream effects (Fig. 6) [292].

### cGAS-STING pathway: DAMP-mediated activation of type I interferon response and the key role of DNA sensors

cGAS functions as a DNA sensor. Upon activation by dsDNA, cGAS catalyzes the synthesis of cGAMP from ATP and GTP. This generated cGAMP, acting as a second messenger molecule, subsequently activates STING through direct interaction. Subsequently, STING translocates from the endoplasmic reticulum (ER) to the Golgi apparatus, where it recruits and phosphorylates TANK-binding kinase 1 (TBK1). TBK1 then phosphorylates and activates both the IRF3 pathway and the NF-κB pathway, triggering the transcription of proinflammatory cytokines, antiviral chemokines, interferon-stimulated genes (ISGs), and type I IFNs. This intricate cascade is known as the classical cGAS-STING signaling pathway (Fig. 6). Furthermore, the cGAS-STING pathway exhibits metal ion dependence; the presence of Mn^2+^ and Zn^2+^ significantly enhances cGAS's sensitivity to dsDNA and increases downstream cGAMP production [265, 266, 293].

The diverse downstream signaling pathways activated by the interactions between DAMPs and PRRs not only operate independently but also establish a dynamic equilibrium via cross-regulation. This intricate interplay jointly governs the complex pathophysiological processes initiated by DAMPs. Therefore, a comprehensive exploration of their specific mechanisms holds the potential to uncover novel therapeutic targets for relevant diseases.

## The role of DAMP in disease pathogenesis

As endogenous danger signals, DAMPs are central to the pathogenesis of numerous diseases, impacting the balance of inflammation, immunity, and tissue repair. DAMPs exhibit complex pathophysiological mechanisms across a range of systemic diseases, including cancers, cardiopulmonary, hepatic, and renal injuries, as well as autoimmune disorders. A selection of DAMP classifications, their corresponding receptors, and associated diseases are summarized in Table 1. Understanding the specific mechanisms by which DAMPs contribute to these diseases holds significant promise for the development of novel therapeutic interventions.

### Cancer

#### Tumor-promoting effects

**Fig. 7:**
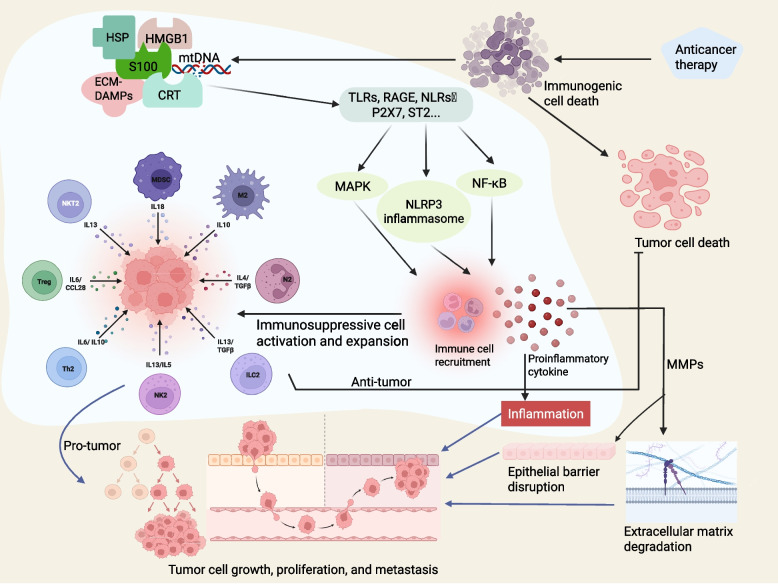
Role of DAMPs in tumor immunity. Immunomodulation and inflammatory response in the tumor microenvironment: immunogenic cell death DAMPs such as HSP, HMGB1, mtDNA, etc., activate receptors such as TLRs, RAGE, NLRs, etc., which in turn triggers pathways such as MAPK, NLRP3 inflammasome NF -κB, etc., to promote the release of pro-inflammatory cytokines and recruitment of immune cells. At the same time, immunosuppressive cells are activated and expanded to secrete cytokines such as IL-10 and TGF-β, creating a complex balance between pro- and anti-tumor. Inflammatory response leads to the destruction of the epithelial barrier and the degradation of the extracellular matrix through MMPs, which ultimately promotes the growth, proliferation and metastasis of tumor cells

Beyond genetic, metabolic, and environmental factors, chronic inflammation is increasingly recognized as a crucial driver in tumorigenesis and tumor progression. Within this context, sterile inflammation, specifically that mediated by DAMPs, plays a pivotal role (Fig. 7) [294]. In the tumor microenvironment (TME), DAMPs released from dying tumor cells activate TLRs. This activation, often involving NF-κB or MAPK signaling, subsequently triggers inflammasome activation, thereby initiating downstream signaling pathways. Concurrently, the IL-1β produced during this process stimulates tumor cells to secrete additional DAMPs, further amplifying inflammation. This sustained inflammatory response, in turn, fosters tumor growth. Furthermore, extracellular matrix (ECM)-derived DAMPs, such as proteoglycans and hyaluronic acid, also influence the inflammation-autophagy balance by regulating the TLR-CD14/CD44 signaling network, which is implicated in tumor development [260, 295–297].

Multiple DAMPs have been shown to promote tumor cell survival, proliferation, invasion, and metastasis [298, 299]. For instance, HMGB1 is strongly associated with cell survival and proliferation and may directly contribute to tumor cell metastasis due to its capacity to enhance cell migration [242]. Specifically, HMGB1 secreted by breast cancer cells promotes fibroblast activation via the RAGE/aerobic glycolysis pathway, and these activated fibroblasts subsequently enhance breast cancer cell metastasis by increasing lactate production [240]. Similarly, IL-1α drives colorectal cancer metastasis by disrupting the epithelial barrier through the upregulation of matrix metalloproteinases and promoting extracellular matrix degradation [281].

Furthermore, certain DAMPs, including HMGB1, S100 proteins, and IL-1α, not only accelerate tumor progression but also contribute to resistance against anticancer therapies [239, 300, 301]. HMGB1, for example, may induce autophagy via the MEK/ERK signaling pathway, which subsequently leads to chemoresistance [241]. The IL-33/ST2 signaling pathway promotes cancer progression and remodels the tumor microenvironment by augmenting immunosuppressive cells [280]. Breast cancer stromal fibroblasts have been observed to shed exosomes containing protein-free/unshielded RNA. These exosomes induce RIG-I signaling in breast cancer cells, ultimately contributing to tumor growth, metastasis, and treatment resistance [298] (Fig. 7).

#### Anti-tumor effects

Immunogenic cell death (ICD) is a form of cancer cell death induced by certain chemotherapeutic agents, physicochemical therapies, photodynamic therapy, and radiotherapy. DAMPs play a crucial role in mediating anti-tumor immune responses triggered by ICD. The DAMPs associated with ICD mainly include HMGB1, ATP, mtDNA, CRT, HSPs, IFNs, and cytokines of the IL-1 family [300]. Studies have demonstrated that the injection of IL-33 into melanoma-bearing mice inhibits tumor progression and prolongs survival, while also reducing lung metastasis in mice with breast cancer [280]. Additionally, HSP70 can stimulate natural killer (NK) cells via CD94 or MICA, leading these NK cells to kill tumor cells either through NKG2D-dependent mechanisms or by increasing the release of granzyme B [302]. In conclusion, when cancer cells undergo ICD, they release DAMPs that are recognized by PRRs, subsequently activating innate and adaptive immune responses. This process promotes effective infiltration of immune cells such as CD8 + T cells and NK cells and facilitates the formation of immunological memory, thereby exerting anti-tumor effects [295, 303, 304].

Tumor-derived DAMP proteins are overexpressed in various tumor types and are strongly correlated with disease severity and poor prognosis [257]. Therefore, DAMPs have the potential to serve as novel biomarkers for disease management. In the future, they could also be valuable for selecting patient-specific therapies and evaluating treatment responses [242]. For example, in breast cancer (BC) patients, HMGB1 expression serves as an effective indicator of the immunogenicity and efficacy of chemotherapeutic agents, and elevated levels of HMGB1 have been associated with improved clinical outcomes in patients receiving neoadjuvant chemotherapy [305].

### Cardiovascular diseases

#### Pro-inflammatory response

**Fig. 8:**
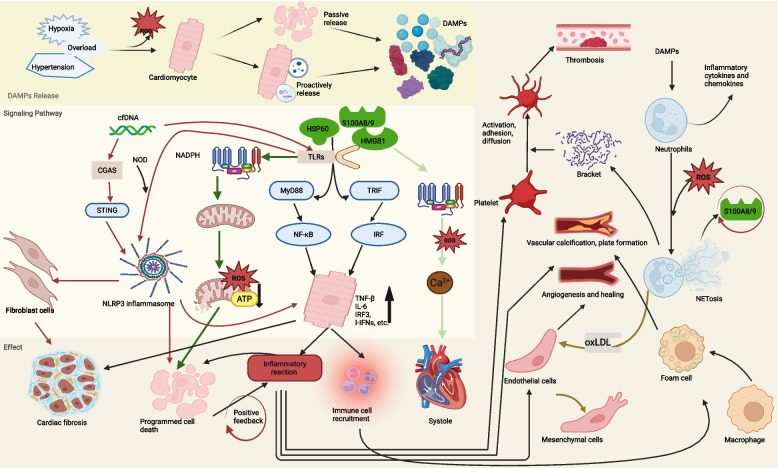
DAMPs—driven cardiac inflammatory injury. Signaling and regulatory mechanisms of DAMPs in cardiovascular pathological processes: hypoxia, overload and hypertension promote DAMPs in cardiomyocytes. these molecules activate downstream pathways via receptors such as cGAS-STING, NOD, TLRs: MyD88 activates NF-κB, triggering inflammatory responses and recruiting immune cells; TRIF activates IRF. meanwhile. cfDNA activates NLRP3 inflammasome, generating ROS and exacerbating programmed cell death. At the same time, cfDNA activates NLRP3 inflammatory vesicles and generates ROS, promoting programmed cell death and exacerbating myocardial fibrosis. On the right side, DAMPs activated platelets to promote thrombosis and vascular calcification, activated neutrophils to produce reactive oxygen species and induced NETosis, and acted on endothelial cells and mesenchymal stromal cells to participate in angiogenesis and foam cell formation

During myocardial infarction (MI), stress conditions such as hypoxia or pressure overload induce cardiomyocytes to release a large number of DAMPs, which bind to pattern recognition receptors, activate innate immunity, and initiate an inflammatory cascade. This process ultimately results in myocardial tissue injury and cardiac dysfunction (Fig. 8). For example, DAMPs such as HMGB1, S100A8/A9, cfDNA, and oxidized low-density lipoprotein (oxLDL) can interact with TLRs, leading to the activation of NF-κB and IRF3 pathways through MyD88-dependent or -independent mechanisms. This activation induces the production of inflammatory mediators such as TNF-α, IL-6, and type I interferons, which further promote inflammatory responses and exacerbate cardiac injury [262, 272, 306–308].

#### Induction of programmed cell death

DAMPs are involved in apoptosis, pyroptosis, and necrosis of cardiomyocytes, which not only leads directly to cardiomyocyte loss, but also further amplifies the inflammatory response and exacerbates myocardial injury and cardiac dysfunction through the release of pro-inflammatory factors and recruitment of immune cells. These effects are mediated through the release of pro-inflammatory factors and recruitment of immune cells. At the site of myocardial infarction, S100A8/A9 binds to TLR4, resulting in impaired mitochondrial function and reduced ATP synthesis, ultimately triggering cardiomyocyte death [262]. Similarly, the release of HSP60 activates TLR4, which, in turn, initiates TNF-α-mediated apoptosis [266]. In the context of myocardial infarction or myocardial I/R injury, DAMPs such as HMGB1, HSPs, mtDNA, and ATP promote cellular focal death by activating the NLRP3 inflammasome, caspase-1, caspase-4/5/11, and GSDMD [272, 309]. Furthermore, during heart failure, cfDNA mediates intercellular communication by interacting with nucleic acid sensors (e.g., TLR9, cGAS) on cell membranes or intracellular compartments, potentially triggering necroptosis and pyroptosis [266].

#### Activation of immune cells

In the early inflammatory phase of MI, HMGB1 promotes the recruitment of neutrophils, monocytes/macrophages, and dendritic cells to the infarct site and stimulates them to secrete inflammatory cytokines, thereby activating the inflammatory response. During the subsequent repair phase, HMGB1 facilitates these cells to transition to a tissue repair phenotype and suppresses the secretion and accumulation of inflammatory factors, ultimately promoting tissue healing [243]. Neutrophils activated by DAMPs not only secrete chemokines and cytokines to regulate inflammation but also promote the formation of NETs through a specialized cell death process called NETosis, which involves oxidative stress. Neutrophils release S100A8/A9 into the extracellular environment via NETosis, and this release further exacerbates neutrophil-mediated inflammation [262, 285, 310]. Additionally, cfDNA and oxLDL contribute to atherosclerosis by activating TLRs and NLRP3 inflammasomes, regulating downstream signaling pathways, and inducing macrophage differentiation into foam cells [306, 311].

#### Fibrosis

In myocardial ischemia–reperfusion injury, DAMPs can induce cardiac fibroblast proliferation through activation of NLRP3 inflammasome, while releasing pro-inflammatory molecules such as IL-1β, IL-6, and TNFa, which in turn exacerbate interstitial fibrosis [266, 272].

#### Myocardial contractile dysfunction

Disruption of calcium homeostasis in the cardiac microenvironment is closely associated with the onset and progression of cardiac disease. DAMPs may influence cardiac function by modulating calcium homeostasis. For instance, HMGB1 can regulate calcium levels through a TLR4/ROS-dependent pathway, leading to calcium leakage from the sarcoplasmic reticulum, a reduction in the amplitude of calcium transients, and decreased cardiomyocyte contractility, which can further aggravate the pathological processes in the heart [307].

#### Oxidative stress

Under hypoxic conditions, ROS can directly damage cardiomyocytes, leading to the release of DAMPs such as HMGB1, ATP, S100A8/A9, and others. These DAMPs activate the innate immune system by binding to pattern recognition receptors, creating a vicious cycle of"inflammatory response → oxidative stress → further release of DAMPs → immune activation" [243, 262, 310]. Additionally, oxidatively modified HMGB1, along with ROS and myeloperoxidase (MPO) released by neutrophils, can promote the formation and rupture of atherosclerotic plaques, thereby accelerating the progression of cardiovascular disease [243].

#### Endothelial cell dysfunction

DAMPs significantly increase the transcription and release of pro-inflammatory factors in endothelial cells by activating TLRs and the NF-κB signaling pathways, thereby triggering chronic inflammation and vascular injury. This persistent inflammatory state further exacerbates endothelial dysfunction and promotes lipid deposition, foam cell formation, and plaque development, contributing to the progression of atherosclerosis (AS) [244]. Not only do NETs and their components (histones, DNA, and myeloperoxidase) directly damage endothelial cells, but their synergistic effects with oxLDL or native LDL further enhance endothelial-to-mesenchymal transition (EndMT), leading to endothelial cell dysfunction and promoting neovascularization, which influences AS progression [285]. Additionally, when mtDNA leaks into the extracellular space from endothelial cells and vascular smooth muscle cells, it can inhibit angiogenesis and wound healing or promote vascular calcification by activating the cGAS-STING-IRF3 signaling pathway, potentially contributing to vascular lesion development [265].

#### Thrombosis

DAMPs also contribute to microthrombosis in the context of atherosclerosis, myocardial infarction, and other conditions. Specifically, HMGB1 regulates platelet activation, granule secretion, adhesion, and spreading via the TLR4 pathway on platelets. S100A8/A9 influences thrombosis by binding to CD36 on platelets. Furthermore, DAMPs indirectly induce thrombus formation through the activation of NETs via the TLR4/9 and RAGE pathways. NETs, in turn, promote platelet adhesion, activation, and aggregation by forming DNA network scaffolds, leading to fibrin deposition and subsequent thrombosis [285, 310] (Fig. 8).

### Lung diseases

#### Pro-inflammatory effects

**Fig. 9:**
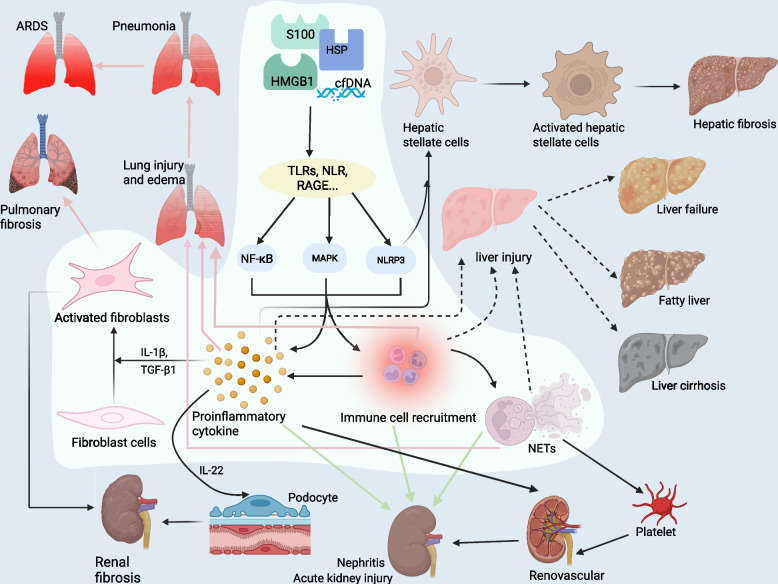
DAMPs—mediated multi—organ inflammatory injury. Schematic diagram of the mechanism of DAMPs in multi-organ pathological processes: This figure demonstrates the mechanism by which molecules such as S100, HMGB1, and HSP activate signaling pathways such as NF-κB, MAPK, and NLRP3 through receptors such as TLRs, NLRs, and RAGE, triggering the release of pro-inflammatory cytokines and recruitment of immune cells, which then mediate the mechanism of multi-organ pathological changes. In the lungs, it can induce acute respiratory distress syndrome, pneumonia, lung injury, pulmonary edema and pulmonary fibrosis; in the liver, it activates hepatic stellate cells, leading to hepatic fibrosis, hepatic failure, fatty liver and cirrhosis, etc.; and in the kidneys, it involves renal fibrosis, nephritis, acute renal injury, and renal vascular lesions. In addition, the effects of neutrophil extracellular traps on platelets and renal vasculature, as well as the effects of IL-22 on podocytes, are shown, presenting a comprehensive picture of the complex regulatory network of these molecules in multi-organ pathologic processes

In many severe inflammatory lung diseases, including coronavirus disease 2019 (COVID-19), a prolonged host inflammatory response is a major contributor to lung injury and eventual failure [246, 270]. DAMPs produced during ALI increase the production of NLRP3 and pro-IL-1β, promote the nuclear translocation of NF-κB, and further activate NLRP3 inflammasomes in lung macrophages. Activated NLRP3 inflammasomes then trigger inflammatory responses and contribute to severe lung inflammation [248]. Additionally, mitochondrial DAMPs—such as mtDNA, N-formyl peptides (fMLP), cardiolipin, and ATP—released during lung injury, induce proinflammatory responses via NF-κB and MAPK pathways (Fig. 9) [273].

#### Activation of immune cells

Some DAMPs function as chemoattractants, directly recruiting neutrophils [248]. SARS-CoV-2-mediated infection of the lung epithelium results in the release of a variety of cytokines and DAMPs from the cells, molecules that induce recruitment of immune cells. With the release of ROS, activation occurs through autophagy, release of NETs and pro-inflammatory mediators, etc. The resulting mechanism leads to lung injury and edema, which in turn leads to interstitial pneumonitis up to acute respiratory distress syndrome [312, 313].

#### Promotion of fibrosis

Multiple DAMPs are elevated in alveolar epithelial cells of patients with idiopathic pulmonary fibrosis (IPF), which may contribute to fibrosis. DAMPs can directly activate pro-fibrotic factors such as IL-1β, TGF-β1, and TNF-α, thereby promoting fibroblast survival and myofibroblast differentiation. HMGB1, ATP, and uric acid further stimulate macrophages to secrete IL-1β and induce pulmonary epithelial cells to release the chemokine CCL2, processes that collectively influence the fibrotic progressio [247, 314] (Fig. 9).

### Diseases of the liver

#### Pro-inflammatory response

DAMPs-induced sterile inflammation is closely associated with liver disease (Fig. 9) [268, 277]. In a mouse model of acetaminophen-induced liver injury, damaged hepatocytes release DAMPs such as HMGB1 and HSP-70, which activate Kupffer cells, promote the production of inflammatory cytokines, and modulate both pro- and anti-inflammatory responses [315]. In nonalcoholic fatty liver disease, endogenous denatured DNA, cholesterol, and HMGB1 derived from apoptotic and necrotic host cells, together with mtDNA, formylated peptides, and ATP released due to mitochondrial dysfunction, induce inflammation by activating the cGAS/STING pathway and NLRP3 inflammasomes [267]. Inflammatory mediators released by immune cells can further stimulate additional cells, generating a positive feedback loop that exacerbates liver injury.

#### Promotion of fibrosis

Fibrosis is a key pathological process in the progression of chronic liver disease to cirrhosis. DAMPs such as HMGB1, IL-33, and ATP released from liver injury bind to TLR2/4 and activate hepatic stellate cells (HSC), prompting their transformation into myofibroblasts and secretion of a large number of extracellular matrix proteins, which leads to matrix deposition and fibrosis [277, 316, 317]. Additionally, DAMPs promote the release of pro-inflammatory factors, such as IL-1β, by mediating the assembly and activation of the NLRP3 inflammasome, thereby indirectly enhancing HSC activation and extracellular matrix accumulation [317]. Fragmented ECM components (e.g., platelet-derived proteins and proteoglycans) generated during tissue remodeling are recognized by immune cells as DAMPs, further amplifying inflammatory and fibrotic signaling via TLR2/4 [318, 319].

#### Induction of programmed cell death

Alcoholic liver disease, decompensated cirrhosis, and pharmacological liver injury all cause sustained hepatocellular and tissue damage, leading to the release of DAMPs such as HMGB1, extracellular histones, and mtDNA. These DAMPs exacerbate liver injury by modulating inflammatory responses and promoting fibrotic processes [249, 320]. The inflammatory responses triggered by DAMPs further contribute to cell death in a vicious cycle; for example, extracellular histones activate TLR2/4-dependent inflammatory pathways that regulate cell death during sterile inflammatory responses [106, 267].

#### Activation of immune cells

During liver injury, DAMPs trigger an inflammatory response via PRRs and inflammasomes, leading to the infiltration of neutrophils, monocytes, and macrophages, as well as the activation of Kupffer cells. This response is further amplified by the release of pro-inflammatory factors, resulting in sustained inflammation that exacerbates tissue damage and may contribute to decompensation [271, 320]. Simultaneously, the binding of DAMPs to PRRs on immune cells also promotes the progression of fibrosis [267] (Fig. 9).

### Kidney diseases

#### Pro-inflammatory effects

In renal diseases, renal non-specific DAMPs such as HMGB1, histones, mtDNA, and uric acid activate PRRs to trigger innate immunity, which in turn mediates tubular inflammatory responses (Fig. 9) [283, 321, 322]. Crystal deposition and urinary regulatory proteins, as kidney-specific DAMPs, are also involved in inducing renal inflammation. Crystals can be taken up by mesenchymal dendritic cells via phagocytosis, a process that triggers the assembly and activation of NLRP3 inflammatory vesicles, leading to the release of IL-1β, which in turn triggers the release of NF-kB-dependent cytokines and chemokines, amplifying the inflammatory response [323]. In distal tubular injury, leakage of urinary regulatory proteins into the interstitial space activates dendritic cells through TLR4 and NLRP3 inflammasomes, initiating an immune response [323, 324].

#### Promoting repair and regeneration

DAMPs participate in the repair and regeneration processes following renal injury. Increased expression and release of HMGB1 in kidneys subjected to unilateral ureteral obstruction (UUO) have been shown to promote interstitial fibrosis by inducing M1 macrophage polarization; conversely, blocking HMGB1 release or counteracting its function alleviates inflammation and fibrosis [251, 278]. In diabetic nephropathy, inhibition of HMGB1 has been observed to attenuate podocyte apoptosis and epithelial-mesenchymal transition [325]. Uric acid (UA), acting as a DAMP, can directly activate TLR4, leading to renal fibrosis, glomerulosclerosis, and elevated levels of inflammatory mediators [283]. Mice deficient in the S100A8/A9 heterodimer are protected from renal fibrosis following UUO treatment, indicating that S100A8/A9 mediates renal fibrosis, tubular apoptosis, and renal epithelial-mesenchymal transition post-UUO [263]. Some DAMPs activate TLR2 on renal progenitor cells, thereby accelerating tubular repair. Moreover, activation of TLR4 on renal dendritic cells by DAMPs triggers the release of IL-22, which stimulates IL-22 receptors on renal tubular epithelial cells (TECs) and promotes tubular re-epithelialization [278].

#### Affecting hemodynamics

In infectious acute kidney injury, necrotic cell-derived DNA induces platelet activation and platelet-granulocyte interactions, which subsequently promote NET formation, thereby exacerbating renal inflammation and tissue injury [279, 322].

#### Activation of immune cells

DAMPs promote immune responses and facilitate the recruitment of immune cells (e.g., neutrophils) to infiltrate injured tissues. Additionally, DAMPs contribute to tubular regeneration and the restoration of renal function through macrophage polarization and interactions with TLR2 and TLR4 [278, 322].

#### Induction of autoimmune response

Some DAMPs serve as important autoantigens that contribute to renal autoimmune diseases. For example, the release of nucleosomes and dsDNA from glomerular cells can promote the binding of lupus autoantibodies to the glomeruli, thereby triggering lupus nephritis [323].

#### Induction of programmed cell death

Injury-induced necrosis results in the release of DAMPs from renal endothelial and epithelial cells. These DAMPs then activate PRRs on renal dendritic cells and macrophages. Subsequently, these effector cells produce inflammatory cytokines and chemokines, which further promote necrosis of renal cells. Additionally, crystal deposits can directly induce cytotoxicity in renal tubular epithelial cells. Extracellular histones also exert direct toxic effects on vascular endothelial cells; however, the molecular mechanisms underlying this process remain unclear [323, 326] (Fig. 9).

### Other

#### Autoimmune diseases

DAMPs function as important inflammatory mediators in various autoimmune diseases, particularly in RA and SLE [256, 269, 274]. Clinical and experimental studies have demonstrated that HMGB1-TLR-dependent pro-inflammatory mechanisms play a pivotal role in the pathogenesis of these conditions [291]. Local concentrations of HMGB1 are elevated in lupus nephritis (LN), and urinary HMGB1 has been shown to distinguish patients with active LN from those with inactive disease and healthy controls [256]. Furthermore, characterization of cfDNA in patients with SLE indicates that higher cfDNA concentrations, increased fragment numbers, and specific fragment length profiles are associated with worse glomerular filtration rates and more severe lupus nephritis [269].

#### Sepsis

Sepsis is a systemic inflammatory response triggered by infection, characterized by the overproduction of pro-inflammatory cytokines (e.g., IL-1β, IL-6, TNF-α), which ultimately leads to cytokine storm and multiple organ dysfunction syndrome (MODS) [254]. Infection-induced extensive immune cell death releases a substantial amount of DAMPs, with different DAMPs involved in various pathological processes during sepsis. For instance, HMGB1 not only participates in regulating the production of inflammatory mediators but also promotes the entry of lipopolysaccharides into cells, thereby triggering pyroptosis, which plays a crucial role in sepsis. Circulating cell-free DNA may influence the prognosis of patients with severe sepsis by promoting the development of DIC. Elevated levels of S100 proteins have also been associated with increased mortality risk in sepsis patients [254, 327, 328].

#### Diseases of the nervous system

In the pathogenesis of neurological diseases, DAMPs exert their effects by activating neuroinflammatory responses [329]. Modulation or inhibition of HMGB1 release significantly reduces brain injury and improves neurological outcomes in various disease models, including ischemia, hemorrhage, trauma, epilepsy, and AD [255, 330]. Mitochondrial DAMPs activate PRRs on glial cell surfaces, leading to the release of pro-inflammatory factors, activation of inflammatory pathways, and exacerbation of mitochondrial damage—creating a vicious cycle of mitochondrial dysfunction and neuroinflammation [276, 331]. Following traumatic brain injury (TBI), large quantities of S100B protein released from astrocytes and damaged neurons act as typical DAMPs. S100B activates microglia by binding to RAGE receptors, which subsequently triggers the activation of NF-κB and MAPK signaling pathways, resulting in the excessive release of pro-inflammatory cytokines such as IL-1β, TNF-α, and other mediators. This cascade ultimately amplifies the neuroinflammatory response and contributes to secondary brain injury [332, 333].

In addition to the diseases mentioned above, DAMPs also play a critical role in the pathogenesis of various other conditions. For instance, in oral diseases, infection with Porphyromonas gingivalis in patients with periodontitis induces the release of HMGB1 from host cells, which promotes osteoclastogenesis, leading to periodontal tissue destruction and bone resorption [334]. In Sjögren's syndrome, DAMPs released from damaged salivary gland epithelial cells initiate inflammatory and autoimmune responses, resulting in a vicious autoimmune cycle [252, 335]. In diabetes mellitus, HMGB1 release induced by a high-glucose environment sustains low-grade inflammation through RAGE receptors, contributing to insulin resistance and further exacerbating metabolic dysfunction associated with the disease [336, 337]. In summary, DAMPs are broadly implicated in the development of numerous diseases. However, their precise mechanisms of action remain incompletely understood. Evidence suggests that certain DAMPs not only promote tissue injury but also participate in tissue repair processes [241, 243], reflecting their complex biological functions. Therefore, further in-depth investigations are warranted to fully elucidate the regulatory mechanisms of DAMPs under different pathophysiological conditions and to explore their potential therapeutic applications.

## Application of DAMPs targeted therapy

In the exploration of therapeutic strategies aimed at DAMPs and their receptors, three principal approaches have emerged as notably promising: (1) the modulation of cell death pathways to curtail DAMP release; (2) pharmacological intervention for the neutralization of DAMPs or the blockade of downstream signaling (e.g., monoclonal antibodies and small-molecule inhibitors); and (3) gene silencing technologies. These strategies have become research foci characterized by high frontier value and potential.

### Blocking cell death pathways to reduce DAMPs release

#### Inhibition of necrotic apoptosis

Research on neurodegenerative diseases and ischemic injury has revealed the therapeutic potential of targeting the RIPK1/RIPK3 signaling pathway (e.g., through the utilization of Necrostatin—1) in alleviating I/R injury and offering neuroprotection. Mechanistically, this strategy suppresses necroptosis, thereby diminishing the release of DAMPs from injured cells. In glaucoma, the progressive degeneration of retinal ganglion cells (RGCs) represents the core pathological mechanism underlying vision loss. Current clinical management predominantly centers on the reduction of intraocular pressure (IOP) to decelerate disease progression, yet it remains ineffective in preventing RGC death. Emerging evidence suggests that glutamate excitotoxicity activates the RIP1/RIP3/MLKL—mediated necroptotic pathway, inducing dose—and time—dependent necrotic damage in R28 cells and murine retinal RGCs. Pharmacological inhibition of this pathway, via the application of the RIP1—specific inhibitor Nec—1 or the RIP3 inhibitor GSK872, significantly reduces neuronal death, preserves the retinal architecture, and normalizes abnormal protein expression. Moreover, these inhibitors mitigate glutamate—induced ROS overproduction and oxidative stress, while modulating the activation of the NLRP3 inflammasome and the subsequent release of pro—inflammatory cytokines (e.g., IL—1β) [338]. In an independent study exploring ischemic brain injury, Nec—1, a selective inhibitor of RIPK1 kinase activity, was found to inhibit RIPK3/MLKL—dependent necroptotic signaling by impeding RIPK1 phosphorylation at Ser166. This inhibitory effect significantly decreased neuronal mortality and alleviated the severity of brain injury. Remarkably, the treatment simultaneously attenuated ischemia—induced IL—1β maturation and inflammatory activation, indicating that the neuroprotective effects of Nec—1 are mediated via the specific blockade of this pathway [339]. Moreover, Nec—1 has been widely utilized as a selective RIPK1 kinase inhibitor in numerous experimental disease models. Its applications encompass a variety of pathological states, such as I/R injury in the brain, heart, and kidney, along with systemic inflammatory response syndrome (SIRS), sepsis, and associated disorders [340]. Although Nec—1 is still in the pre—clinical phase, its proven efficacy in multiple disease models offers a compelling basis for the clinical translation of targeted modulation of necroptosis/apoptosis. Nevertheless, additional research is necessary to comprehensively assess the specificity and safety of pathway regulation, which will be pivotal for promoting the transformation of these therapeutic strategies from the laboratory to the clinic.

#### Regulating focal deaths

Numerous preclinical investigations have confirmed that the caspase—1 inhibitor VX765 exhibits pleiotropic protective impacts across a spectrum of inflammation—related pathologies, such as HIV infection. Acting as a crucial regulator of the NLRP3 inflammasome—caspase—1—coagulation axis, VX765 predominantly exerts its therapeutic efficacy via caspase—1 inhibition, thus preventing: (1) the assembly of the inflammasome complex, (2) the maturation and secretion of pro—inflammatory cytokines (especially IL—1β and IL—6), and (3) GSDMD—mediated pyroptotic cell death. These mechanistic actions have been consistently verified in various disease models, including atherosclerosis, intestinal ischemia—reperfusion injury (IIRI), and pulmonary ischemia—reperfusion injury (LIRI) [341–343]. Furthermore, VX—765 exhibited cardioprotective effects through dual mechanisms in a rat model of acute myocardial infarction (AMI) [344]. Firstly, VX—765 inhibits the activation of the IL—1β/p38 MAPK signaling pathway, significantly up—regulating the expression of Cx43 (a cardiomyocyte gap junction protein) and directly suppressing the transcriptional activation of pro—inflammatory factors. Secondly, VX—765 specifically blocks the assembly of NLRP3 inflammasomes, reducing caspase—1—dependent pyroptosis, thereby preventing the collapse of mitochondrial membrane potential and the dysfunction of ATP synthesis. By inhibiting the cleavage of GSDMD protein, it maintains the structural integrity of the mitochondrial membrane and reduces the release of harmful signals, such as mtDNA. Consequently, VX—765 suppresses the cascade of mitochondria—derived DAMPs at the origin. Notably, when combined with the platelet inhibitor cangrelor, VX—765 significantly reduces the size of myocardial infarction [345]. Preclinical investigations have indicated that SAMHD1, a myeloid—specific restriction factor, inhibits HIV replication through the degradation of intracellular deoxyribonucleoside triphosphates (dNTPs), thus maintaining dNTP concentrations below the threshold necessary for HIV—1 reverse transcription. Conversely, the HIV—2—encoded accessory protein Vpx counterbalances this restriction by facilitating ubiquitin—mediated degradation of SAMHD1, effectively nullifying its antiviral activity. Significantly, VX—765 exerts a distinctive synergistic effect by suppressing inflammasome—associated caspase—1 activity, which averts pro—inflammatory signal—induced destabilization of SAMHD1. As a result, VX—765 indirectly sustains SAMHD1 protein levels and antiviral function, implying its potential as a novel therapeutic approach for HIV—1 infection. These discoveries emphasize the stabilization of SAMHD1 as a promising target for HIV treatment [346, 347].

### Neutralization of DAMPs and their receptors by monoclonal antibodies

#### Anti-HMGB1 antibodies

In a rat model of hemorrhagic brain injury, the intravenous administration of anti-HMGB1 monoclonal antibody exhibited multimodal protective effects on the integrity of the BBB. Mechanistically, the antibody not only neutralized extracellular HMGB1 to mitigate its cytotoxic effects on cerebrovascular endothelial cells but also inhibited subsequent neuroinflammatory responses [348]. Moreover, the antibody notably inhibited the expression of pro—inflammatory mediators and alleviated the over—activation of glial cells, thereby effectively disrupting the HMGB1—initiated inflammatory cascade in the acute phase of injury [349, 350]. Notably, treatment with HMGB1 monoclonal antibody exhibits dual therapeutic effects: (1) inhibiting neuroinflammation, and (2) reducing the release of active HMGB1 from neurons and its intracellular translocation, thus disrupting the HMGB1-mediated inflammatory positive-feedback loop. Importantly, this therapeutic strategy demonstrated comparable efficacy in a spinal cord injury model, indicating its wide applicability in central nervous system (CNS) injuries [351, 352]. Pretreatment of a murine model with this antibody prior to neural stem cell transplantation substantially promotes motor function recovery subsequent to spinal cord contusion. Clinical investigations suggest that the cerebrospinal fluid (CSF) levels of HMGB1 in patients with subarachnoid hemorrhage (SAH) demonstrate a positive correlation with pro—inflammatory factors, such as IL—6 and TNF—α, and that the degree of HMGB1 elevation is closely related to the severity of the disease [353]. Animal research further indicated that the nuclear translocation of HMGB1 and its subsequent release from the smooth muscle cells of the basilar artery in rats with subarachnoid hemorrhage (SAH) were associated with a persistent vasoconstrictive reaction [354]. Intervention using an anti-HMGB1 monoclonal antibody notably alleviated the severity of vasospasm and inhibited inflammatory responses within the arterial vascular wall subsequent to subarachnoid hemorrhage (SA).

To expand the therapeutic potential of this approach for neurodegenerative diseases, Fujita et al. identified a notable up—regulation of HMGB1 in both patients with AD and APP/PS1 transgenic mouse models via gene expression profiling. Their findings imply that HMGB1 might activate PKC isoforms and aggravate the pathological aggregation of β—amyloid peptide (Aβ). The chronic administration of an anti—HMGB1 monoclonal antibody effectively mitigated Aβ deposition and improved cognitive deficits in this model [355]. In a rat model of Parkinson’s disease (PD) induced by 6-hydroxydopamine (6-OHDA), the systemic administration of the antibody significantly mitigated the degeneration of dopaminergic neurons through the suppression of neuroinflammation, the reduction of ROS generation, and the preservation of BBB integrity [356]. Santoro et al. further demonstrated that an anti-HMGB1 neutralizing antibody could significantly attenuate MPTP-induced dopaminergic neuronal degeneration [357].

#### Others

C5a, a crucial pathological mediator in the early stage of sepsis, triggers excessive inflammatory responses. When its level remains persistently elevated, it can result in immune cell apoptosis. Animal studies employing C5aR1—deficient mice have shown notably enhanced survival rates in mild—to—moderate sepsis models. Remarkable therapeutic progress has been made in targeting the C5a pathway, as exemplified by vilobelimab (IFX—1), a humanized IgG4 monoclonal antibody. In a German multicenter phase IIa clinical trial, vilobelimab demonstrated both a satisfactory safety profile and a dose—dependent improvement in organ dysfunction among patients with severe sepsis, while maintaining normal membrane attack complex (MAC) formation [358].

In research exploring atherosclerosis, a radiolabeled monoclonal antibody F(ab')_2_ fragment directed against the V—domain of RAGE was developed. The uptake intensity of this tracer in atherosclerotic lesions was discovered to demonstrate a significant correlation with RAGE expression and macrophage infiltration in both diabetic and non—diabetic ApoE < sup > -/- </sup > mouse models [359]. In comparison to conventional inflammation imaging techniques, this approach facilitates not only the dynamic surveillance of inflammatory activity but also the specific recognition of the AGEs—RAGE pathway triggered by chronic hyperglycemia. These discoveries offer both imaging evidence and pathological backing for targeting RAGE in the diagnosis and treatment of diabetic atherosclerosis.

Although monoclonal antibodies targeting DAMPs and their receptors have achieved remarkable progress in therapeutic research, several challenges still persist for clinical translation. Firstly, large-scale production and cost-effectiveness pose substantial obstacles to the extensive clinical application. Secondly, the intrinsic immunogenicity of monoclonal antibodies may trigger anti-drug antibody (ADA) responses, which could potentially undermine therapeutic efficacy and lead to adverse reactions. Moreover, optimal target selection and treatment timing strategies need to be formulated to maximize therapeutic benefits while minimizing side effects.

### Enzymatic degradation of DAMPs

In diseases characterized by inflammation mediated by DAMPs, enzymatic degradation strategies exhibit substantial clinical potential through the targeting and clearance of pathogenic molecules. Urate oxidase agents (e.g., rasburicase, pegloticase) promptly lower serum urate levels in gout and tumor lysis syndrome by catalyzing the transformation of uric acid into soluble allantoin [360]. Rasburicase is currently authorized for the treatment of acute hyperuricemia. The findings from its phase IV trial (NCT05312268) indicated that the combination of rasburicase with oral urate—lowering medications achieved short—term normalization of serum urate levels. Nevertheless, the long—term efficacy of rasburicase is restricted by its immunogenicity and short half—life. Pegloticase, a pegylated urate oxidase, features an extended half—life of 14 days and a decreased incidence of allergic reactions, and it has been approved for the management of refractory gout. However, a reduction in efficacy is observed in some patients due to the development of antibodies.

DNA enzymes (e.g., DNase I) demonstrate distinctive mechanisms and clinical prospects in the treatment of lupus nephritis (LN). Their fundamental function entails the degradation of extracellular DNA and NETs, which reduces the deposition of immune complexes and subsequently mitigates renal inflammation. Research suggests that the activity of renal DNase I is notably decreased in LN patients, which impairs the clearance of NETs. The supplementation of exogenous DNase I effectively reduces the deposition of renal immune complexes and inhibits the NF—κB pathway as well as the release of inflammatory cytokines [361]. Clinical trials demonstrate that recombinant human DNase I (rhDNase) exhibits a favorable safety profile among patients with lupus nephritis (LN). However, it does not lead to a significant improvement in serum anti—double—stranded DNA (anti—dsDNA) antibody levels. This implies that monotherapy might be inadequate to reverse systemic immune dysregulation [362]. Clinical application challenges encompass a brief half—life and suboptimal delivery efficiency. Moreover, the data from phase II or III clinical trials of DNase I in lupus nephritis (LN) are inadequately disclosed, and its long—term safety and synergistic effects with other medications necessitate validation. Future research ought to concentrate on the translation of targeted delivery technologies and the optimization of combination therapies to promote the practical implementation of DNase I in the treatment of LN.

### Blocking DAMP receptors and signaling pathways

#### TLR inhibitors

Research on TLR inhibitors has revealed extensive potential for disease intervention during both pre—clinical and clinical trial phases. Among these inhibitors, the TLR4 inhibitor TAK—242 exhibits promise in the treatment of inflammatory diseases, including sepsis and acute lung injury, by obstructing the LPS—or HMGB1—mediated signaling pathways. Findings from animal studies have corroborated that TAK—242 effectively alleviates LPS—induced acute lung injury, inhibiting neutrophil infiltration and the activation of the nuclear factor kappa—B (NF -κB) pathway [363]. Recent research also reveals that TAK-242 protects hepatocyte mitochondrial function by inhibiting the TLR4/RIPK1 pathway [364]. A present randomized, double—blind, placebo—controlled multicenter phase IIa trial is assessing the efficacy and safety of intravenous TAK—242 in patients with acute alcoholic hepatitis (NCT04620148). The synthetic glycolipid FP7, functioning as a TLR4 inhibitor, diminishes TNF—α levels and enhances hemodynamic parameters in septic shock models, indicating therapeutic potential [365]. JKB-122, a TLR4 antagonist, exhibits hepatoprotective effects in autoimmune hepatitis by inhibiting the production of cytokines including IL-6, IL-17A, and TNF-α [366]. In a phase II trial (NCT02556372) involving patients with refractory autoimmune hepatitis (AIH), JKB—122 has demonstrated favorable outcomes accompanied by satisfactory safety profiles. In investigations regarding hepatic ischemia—reperfusion injury, Eritoran, a crucial TLR4 antagonist, mitigates liver inflammation and cellular impairment through the blockade of the HMGB1—TLR4 axis [367].

The TLR3 antagonist SMU—CX24 exhibits notable efficacy in the treatment of atherosclerosis. Through the inhibition of the TLR3 signaling pathway, it diminishes the uptake of oxidized low—density lipoprotein by macrophages, thereby effectively suppressing the formation of foam cells and the release of inflammatory cytokines. In apolipoprotein E knockout (ApoE^-^/^-^) mouse models, it significantly reduces the area of atherosclerotic plaques and the formation of lipid necrotic cores, concurrently decreasing serum inflammatory factors (e.g., interleukin—6, tumor necrosis factor—α) [368].

Furthermore, inhibitors of the CU—CPT series targeting TLR8 impede signaling pathways by stabilizing TLR8 dimers, which verifies their therapeutic potential for autoimmune diseases in transgenic mice. This discovery offers novel directions for the development of TLR8—targeted drugs, despite the fact that the clinical translation of these findings is still in its nascent stage [369]. The TLR7/8/9 inhibitor R406 suppresses B-cell hyperactivation by blocking Syk kinase activity, showing efficacy in SLE and rheumatoid arthritis preclinical models [369, 370]. In pancreatitis models, the TLR9 antagonist COV08-0064 inhibits neutrophil activation and improves pancreatic pathology [371]. Specific inhibitors IMO—3100 (a TLR7/9 antagonist) and IMO—8400 (a TLR7/8/9 antagonist) markedly inhibit the IL—23—induced expression of IL—17A. In the pathogenesis of psoriasis, they curtail the expression of IL—17A and chemokines through the modulation of the IL—23/Th17 axis [372]. A Phase II trial of IMO—3100 in psoriasis (NCT01622348) indicated that, subsequent to treatment, the mRNA levels of IL—17A and CXCL1 in lesional tissues were significantly downregulated, and the proportion of Th17 cells was reduced. This corroborates that IMO—3100 effectively impedes the IL—23/Th17 inflammatory cascade by inhibiting TLR7/9 signaling. The study offers clinical evidence for targeting the endogenous nucleic acid—TLR7/9 pathway in the treatment of psoriasis. No severe adverse events were observed, which supports the conduct of further Phase III trials to validate long—term efficacy and safety. In a Phase IIa psoriasis trial, IMO—8400 exhibited superior clinical improvement compared to conventional therapies [373]. In the realm of hepatocellular carcinoma (HCC) research, the inhibition of TLR7/9 by IRS—954 or chloroquine demonstrates potential efficacy. This is achieved through the down—regulation of the TLR7/MyD88/NF -κB pathway, which in turn reduces M2 macrophage infiltration or enhances chemosensitivity. These findings imply the existence of novel therapeutic strategies [374, 375].

These investigations clarify the anti—inflammatory mechanisms of TLR antagonists in various diseases and offer crucial bases for clinical translation. Nevertheless, the long—term efficacy and safety of certain agents necessitate further exploration.

#### RAGE inhibitors

FPS-ZM1, an inhibitor of the RAGE, is currently in the pre—clinical research phase and has exhibited promising therapeutic efficacy in numerous animal studies. In primary rat microglia, FPS—ZM1 suppressed AGEs—induced neuroinflammation and oxidative stress by significantly inhibiting the overexpression of RAGE, RAGE—dependent microglial activation, and the nuclear translocation of NF—κB p65. Moreover, it decreased the production of downstream inflammatory mediators, such TNF—αand IL—1β, while alleviating AGEs—stimulated nicotinamide adenine dinucleotide phosphate (NADPH) oxidase activation and ROS generation [376]. In a demyelinating murine model, FPS—ZM1 alone was unable to reverse the impaired proliferation and differentiation of neural stem cells (NSCs), yet it restored dendritic and spine development in the demyelinated hippocampus. Nevertheless, when combined with CRTH2 inhibitors, FPS—ZM1 not only alleviated the deficits in NSC proliferation and differentiation induced by demyelination but also further promoted dendritic and spine development. Additionally, this combinatorial therapy significantly ameliorated cognitive function [377]. In a rat model of focal cerebral ischemia, FPS-ZM1 attenuated neuroinflammation through inhibition of the RAGE/DIAPH1 signaling pathway [378]. In an aged rat model of isoflurane-induced anesthesia, FPS-ZM1 mitigated postoperative cognitive dysfunction (POCD). This protective effect might be mediated via the suppression of Aβ deposition in the hippocampal CA1 region [379].

Azeliragon (TTP488), an orally—administered small—molecule antagonist of the RAGE, can effectively traverse the blood—brain barrier. It has demonstrated promising therapeutic potential in preclinical and clinical investigations regarding AD and diverse cancers. Preclinical evidence indicates that it alleviates the pathological progression of AD through multiple mechanisms. In the TgAPP/PS1 mouse model, azeliragon curtails the deposition of amyloid—beta (Aβ) oligomers by impeding the binding of Aβ to RAGE. It also reduces the release of pro—inflammatory factors by blocking the MAPK/ERK pathway activated by S100 calcium—binding protein A8/A9 (S100A8/A9) and HMGB1. Moreover, it enhances hippocampal perfusion by ameliorating RAGE—mediated vascular endothelial dysfunction. These effects were consistently observed in animal models, significantly retarding cognitive decline and improving cerebral blood flow [380, 381].

#### Inflammatory vesicle inhibitors of NLRP3

MCC950 is a highly selective inhibitor of the NLRP3 inflammasome, which specifically targets the NACHT domain of the NLRP3 protein, thereby disrupting its interaction with ASC. This mechanism effectively suppresses the assembly of the inflammasome and the subsequent secretion of IL—1β and IL—18. Preclinical investigations have revealed the extensive therapeutic potential of MCC950 across multiple disease models. In an angiotensin II (Ang II)—induced aortic aneurysm model, MCC950 significantly reduced the incidence of aneurysms by suppressing macrophage infiltration and preventing collagen degradation. Likewise, in an aortic coarctation model, MCC950 effectively inhibited the activation of the NLRP3/caspase—1/IL—1β pathway, resulting in a decreased incidence of coarctation and mortality. Moreover, it attenuated the phenotypic transformation of smooth muscle cells and alleviated elastic fiber fragmentation [382]. Moreover, investigations within a porcine myocardial infarction model have shown that MCC950 notably diminishes infarct size and alleviates cardiomyocyte pyroptosis through the inhibition of NLRP3 inflammasome activation, consequently reducing the release of inflammatory factors (IL—1β, IL—18). Additionally, MCC950 enhances mitochondrial function by restoring membrane potential and augmenting ATP production [383]. In experimental autoimmune encephalomyelitis (EAE) models, MCC950 effectively suppresses microglial activation and Th17 cell differentiation, consequently attenuating disease progression [384]. In a lung cancer model, MCC950 was found to reduce IL-1β secretion, suppress angiogenesis, and inhibit metastasis through NLRP3 pathway inhibition in tumor-associated macrophages (TAMs) [383]. In clinical research, MCC950 has been approved for the treatment of Cryopyrin—Associated Periodic Syndromes (CAPS). Phase III trials, such as the CANTOS trial, have verified its efficacy in reducing high—sensitivity C—reactive protein (hs—CRP) levels and alleviating the risk of major adverse cardiovascular events (MACE) in patients with cardiovascular diseases [382].

Dimethyl sulfoxide (DMSO), a non—selective inhibitor of NLRP3, exerts its inhibitory function by disrupting the interaction between NLRP3 and thioredoxin—interacting protein (TXNIP). This disruption impedes NLRP3 oligomerization and the subsequent formation of downstream ASC speckles, thereby ultimately obstructing inflammasome assembly and activation [385]. The clinical application of DMSO remains limited, with interstitial cystitis as its only approved indication, while its use in other fields is still in the exploratory stage.

#### JAK-STAT pathway inhibitors

The central role of JAK-STAT signaling in maintaining immune homeostasis provides a strong rationale for targeting this pathway in the treatment of autoimmune and inflammatory disorders [386]. Subsequent to the revelation of the therapeutic potential of JAK inhibitors in the 1990 s, the United States Food and Drug Administration (US FDA) expeditiously sanctioned the first small—molecule JAK inhibitors. These included ruxolitinib for the treatment of myeloproliferative neoplasms (e.g., polycythemia vera and primary myelofibrosis) and tofacitinib for RA. Subsequently, tofacitinib was also approved by the European Medicines Agency (EMA). As the inaugural JAK inhibitor authorized for autoimmune diseases, tofacitinib predominantly inhibits JAK1 and JAK3, exerts partial inhibitory activity against JAK2, and has a minimal impact on TYK2 [387, 388]. Moreover, it is formulated into an extended—release dosage form through osmotic pump technology, which allows for once—daily administration. When compared with the immediate—release (IR) formulation, the extended—release (XR) version shows equivalent systemic exposure, yet it presents a prolonged time to reach the peak concentration and an extended elimination half—life [389]. Despite demonstrating efficacy in Phase III clinical trials, tofacitinib failed to receive FDA approval for the oral treatment of psoriasis owing to safety concerns associated with higher dosage regimens [390, 391]. Clinical trials have demonstrated that tofacitinib exhibits differential efficacy in inflammatory bowel diseases. While the drug showed significant therapeutic effects in phase II/III induction therapy for ulcerative colitis, it failed to demonstrate clinical efficacy in phase II trials for Crohn's disease [392]. Ruxolitinib targets JAK1/JAK2 (with moderate inhibition of TYK2) [393] and is approved for true erythrocytosis and intermediate- to high-risk primary myelofibrosis [394, 395] and showed potential to improve ACR response and HAQ-DI in a phase IIa trial in RA [396]; baricitinib, a selective JAK1/JAK2 inhibitor, is effective in ulcerative colitis (UC) by blocking prokinetic factors such as IL-6, IL-12, IL-23 and IFN-γ. and IFN-γ by blocking intracellular signaling of pro-inflammatory factors such as IL-6, IL-12, IL-23, and IFN-γ [397], significantly improved Disease Activity Score (DAS28-CRP), HAQ-DI, and patient-reported pain, dysfunction, and ruxolitinib demonstrated significant improvement in fatigue symptoms in Phase III RA clinical trials, including the RA-BEAM and RA-BUILD studies [398, 399], furthermore, in a Phase IIb clinical trial, ruxolitinib demonstrated significant efficacy in plaque psoriasis treatment, as evidenced by improved Psoriasis Area and Severity Index 75 response rates [399].

The adverse effect spectrum of JAK inhibitors directly results from their mechanism of action in obstructing the JAK—STAT signaling pathway. In RA clinical trials, nasopharyngitis and upper respiratory tract infections are the most frequently reported adverse events. More notably, these agents have been linked to opportunistic infections, such as herpes zoster, tuberculosis, and specifically in renal transplant recipients at higher doses—BK virus—associated nephropathy [400–402]. Hematologic abnormalities, such as neutropenia and anemia, are infrequently observed during JAK inhibitor therapy. Nevertheless, mild—to—moderate cytopenias exhibit a dose—dependent prevalence, with higher incidence rates being noted in patients administered high—dose tofacitinib regimens [403]. In light of the aforementioned safety considerations, the FDA has issued warnings regarding drugs like tofacitinib, highlighting the risks associated with cardiovascular events, thrombosis, and malignancies. During the clinical application of these drugs, it is imperative to strike a balance between efficacy and risks, and to conduct regular monitoring of blood routine, infection indicators, and liver and kidney functions.

Given this context, the next generation of highly selective JAK inhibitors has emerged. Filgotinib exhibits 30-fold greater selectivity for JAK1 over JAK2 [397], In phase II studies for RA (NCT01888874, NCT01888874), higher daily doses effectively controlled disease activity, demonstrating superiority over placebo both as monotherapy and in combination therapy. Moreover, in the FITZROY trial (NCT02048618) for moderate—to—severe Crohn's disease (CD), it notably enhanced clinical disease activity indices and the quality of life of patients. ABT—494 attains 74—fold greater selectivity for JAK1 over JAK2 by targeting both the ATP—binding site in the JH1 domain and an outer region of JAK1. Its phase IIb studies (NCT02706873, NCT02706951) demonstrated that among patients with moderate—to—severe RA who had an insufficient response to methotrexate (MTX) or anti—TNF therapy, the treatment groups achieved significantly higher ACR 20/50/70 response rates and more substantial improvements in DAS28—CRP scores compared to the placebo group, with a rapid onset of efficacy [404]. Its ongoing phase III study (NCT02629159) is comparing its efficacy to adalimumab against a background of stable MTX dosing.

#### cGAS-STING pathway inhibitors

The cGAS—STING pathway assumes a crucial position within the innate immune system. Nevertheless, its abnormal over—activation gives rise to an inflammatory microenvironment, leading to damage of healthy tissues, cell demise, and organ pathological changes. This dysregulation is associated with a variety of disorders, such as Aicardi—Goutières syndrome (AGS), STING—associated vasculopathy with infantile onset (SAVI), ulcerative colitis, non—alcoholic fatty liver disease (NAFLD), and amyotrophic lateral sclerosis (ALS) [405–410]. The development of inhibitors targeting this pathway has emerged as a prominent focus in both biomedical research and therapeutic development.

Despite substantial enthusiasm, the advancement of cGAS inhibitors is still in an initial phase. Current methodologies predominantly fall into two classifications: catalytic site inhibitors and DNA—binding inhibitors. Catalytic site inhibitors operate by emulating substrate binding to impede cGAS enzymatic activity. For example, in biochemical assays, PF—06928215 manifested binding ability, yet it failed to display inhibitory effects in cellular assays [411]; RU—521 binds to the catalytic site of cGAS and selectively inhibits the dsDNA—dependent enzymatic activity in both in vitro and cellular experiments [412]; The G150 series compounds targeting the ATP/GTP-binding pockets of human cGAS demonstrate potent inhibitory activity in THP-1 cells [413], and compound S3, identified through molecular dynamics simulations and virtual screening, exhibits a mechanism of action analogous to PF-069282155 [414]; The latter inhibits cGAS activation through competitive binding to DNA. For instance, the antimalarial agent hydroxychloroquine (HCQ) suppresses cGAS by binding to the DNA groove of the cGAS—DNA complex [415]; The natural monoterpene compound perilla aldehyde (PAH) disrupts the binding of cGAS to dsDNA [415]; Sulforaphane inhibits cGAS activity by dose-dependently blocking the binding of dsDNA-cGAS [416]; And aspirin inhibits its own activity by acetylating the K384, K394, and K414 residues of cGAS. The acetylation of K414, in particular, suppresses autoimmune responses triggered by self-DNA [417].

The advancement of STING antagonists has advanced at a relatively sluggish pace. H—151, a covalent STING inhibitor, impedes STING palmitoylation and its interaction with TBK1, exhibiting efficacy across multiple disease models [418–420]. Nitrofurans (e.g., C—176) and indolylureas (e.g., H—151), initially reported in 2018, covalently modify the C91 residue of human STING. This modification blocks the oligomerization and palmitoylation processes, ultimately inhibiting the activation of STING. Likewise, the recently discovered BB—Cl—amidine covalently targets the C148 residue of STING, demonstrating a similar inhibitory mechanism. Both types of compounds suppress STING activation via the covalent modification of C91, which disrupts the oligomerization and palmitoylation of STING [421]. Recent research has demonstrated that BB—Cl—amidine exerts its inhibitory function through the covalent modification of the C148 residue of STING, utilizing a mechanism similar to that of previously reported compounds [422].

Nevertheless, to date, nearly all reported STING antagonists necessitate frequent high—dose intraperitoneal administration due to their rapid systemic clearance (for instance, H—151 demonstrates a circulation half—life of less than 2 h after injection). This administration approach poses substantial clinical challenges: it is not only unfeasible for therapeutic applications, but systemic STING inhibition may also undermine antipathogen immunity and tumor immunosurveillance, potentially enhancing patients'vulnerability to infections and cancer [423–425]. To surmount these limitations, researchers are devising STING pathway inhibitory nanoparticles (SPINs) founded on polylactic acid—glycolic acid copolymers (PLGA). These nanoparticles capitalize on the high biocompatibility and degradability of PLGA to facilitate the targeted delivery of RU.521 and H—151 to inflammatory cells. In vitro investigations illustrate that SPINs efficiently suppress the activation of cGAS/STING in macrophages, curbing the production of IFN—I and impeding STING—mediated M1 polarization. This approach constructs a modular and sustainable platform for the targeted treatment of STING—related diseases while minimizing systemic adverse effects [426–428].

Notable progress has been made in preclinical investigations, where numerous inhibitors have shown therapeutic potential for STING—related diseases in animal models. Nevertheless, clinical translation is still in its nascent stage and encounters considerable challenges, such as sub—optimal pharmacokinetic profiles, safety issues, and the requirement for precise therapeutic approaches. Future endeavors ought to center on the structural optimization of inhibitors to improve their pharmacokinetic and physicochemical characteristics, along with the development of safer and more efficient delivery techniques. With the continuous advancement of research, cGAS—STING inhibitors are promising for bringing about novel therapeutic breakthroughs in inflammatory disorders, autoimmune diseases, and certain malignancies.

### Gene silencing technology

Gene silencing technology, as a potent approach for the precise regulation of gene expression, assumes a crucial role in targeting DAMPs and their receptors. Specifically, RNA interference (RNAi) utilizes small interfering RNA (siRNA) or short hairpin RNA (shRNA) to precisely bind to the messenger RNA (mRNA) of DAMP receptor genes via base-complementary pairing. This binding triggers mRNA degradation, thereby reducing the expression levels of the receptor genes and subsequently decreasing the synthesis of receptor proteins. This technique is relatively straightforward and cost-efficient, and has been extensively applied in experimental research. For instance, research has demonstrated that the mRNA and protein expression levels of HMGB1 are notably higher in bladder cancer (BUC) tissues and cell lines compared to non-tumor cells. This finding emphasizes the pivotal role of HMGB1 in BUC progression. When a specific HMGB1 shRNA plasmid was constructed and transfected into BUC cells, RNAi technology successfully silenced HMGB1 expression, which significantly suppressed cell proliferation and induced apoptosis. Additionally, it led to a cell cycle arrest at the G0/G1 phase and a reduction in the number of cells in the S phase. Further exploration revealed that the downregulation of HMGB1 expression effectively inhibited the NF-κB/p65 and its binding activity to DNA, thereby reducing the expression of vascular endothelial growth factor C (VEGF-C) and impeding tumor angiogenesis. Moreover, the activity of matrix metalloproteinase-2 (MMP-2) and MMP-9 was significantly downregulated, and the expression of proteins related to epithelial-mesenchymal transition (EMT) was altered. These alterations collectively resulted in a significant suppression of BUC cell migration and invasion [429].

Conversely, CRISPR—Cas9 technology is capable of attaining permanent silencing of target genes via precise gene editing. A precise gene—editing strategy founded on CRISPR—Cas9 has been successfully devised to efficiently knock down the NLRP3 gene in macrophages. This is accomplished with the assistance of an optimized polymer nanoparticle delivery system named CLAN. This strategy effectively obstructs the secretion of IL—1β and IL—18, along with the activation of caspase—1, thereby suppressing cell death and the NF—κB pathway. It has been proven to exert dose—dependent anti—inflammatory effects in models of sepsis, peritonitis, and type 2 diabetes (T2D) [430]. In other disease models, this technology has also exhibited distinctive advantages. For instance, in a model of LPS-induced acute liver injury utilizing CRISPR-Cas9 TLR4 knockout mice, the deletion of the TLR4 gene inhibited the TLR4/MyD88/NF-κB pathway, diminished the secretion of pro-inflammatory cytokines such as TNF-α and IL-1β, and decreased the expression of apoptosis-associated proteins in hepatocytes [431, 432]. In AD model, the knockdown of the NLRP3 gene mediated by CRISPR—Cas9 notably diminished amyloid—β (Aβ) deposition through the inhibition of inflammasome activation and efficiently curbed microglial hyperactivation [433].

Nevertheless, these two core gene silencing technologies exhibit certain limitations when targeting DAMPs and their receptors. Although RNAi is straightforward and cost—efficient, it has a relatively brief duration of action and is susceptible to off—target effects, which may undermine the accuracy and reliability of experimental outcomes. To surmount these challenges, diverse optimization strategies have been put forward. These encompass the utilization of chemically modified siRNAs, such as cholesterol—conjugated siRNAs, the employment of viral vectors (e.g., lentiviruses) to prolong the duration of action, and the combination with lipid nanoparticles (LNPs) to improve delivery efficiency [434, 435]. In contrast, CRISPR—Cas9 is capable of attaining permanent gene silencing; however, it entails the risk of off—target effects, potentially leading to non—specific DNA cleavage at other genomic loci and presenting potential immunogenicity concerns. To rectify these deficiencies, the design of guide RNA (gRNA) can be optimized to strengthen its specific binding to target genes, or base editing technology can be utilized to diminish non—specific DNA cleavage [436, 437].

In practical disease modeling applications, RNAi is more appropriate for short—term inflammation regulation because of its rapid onset of action. For instance, during the acute phase of sepsis, it can promptly suppress the inflammatory response [438]. In contrast, the CRISPR—Cas9 system is more suitable for long—term disease intervention. For example, in the treatment of neurodegenerative diseases, this is attributed to the long—term stability of gene modification [439]. In the future, it is anticipated that combined therapeutic strategies will emerge as a research focal point. For example, RNAi can be employed to promptly suppress acute inflammation and manage the disease during its early phases, followed by the utilization of the CRISPR—Cas9 system to impede the long—term pathological advancement of the disease. Alternatively, dual—target editing strategies can be implemented, such as concurrently silencing HMGB1 and RAGE, to augment therapeutic effectiveness and offer novel solutions for surmounting complex diseases (Table 2).

**Table 2:** Therapeutic strategies targeting DAMPs: mechanisms and clinical development

Classification of Drugs	Name of Drugs	Target/Mechanism of Action	Clinical trial phase and number	Indications	Bibliography
Blocking cell death pathways	Necrostatin-1 (Nec-1)	Mutational inhibitor of RIPK1 kinase activity, inhibits RIPK1 phosphorylation at Ser166 residue, blocks RIP3/MLKL-dependent necroptotic apoptotic signalling, and reduces the release of DAMPs such as HMGB1, mtDNA, etc	Preclinical	Ischaemia–reperfusion injury (brain, heart, kidney), neurodegenerative diseases (glaucoma, Parkinson's disease), sepsis	[338–340]
	VX765	Caspase-1 inhibitor, blocks the NLRP3 inflammasome-caspase-1-effector axis, inhibits caspase-1 activity and GSDMD-mediated cellular cellular cell death, and reduces the release of pro-inflammatory factors such as IL-1β and IL-6	Phase II NCT01048255 NCT00205465 NCT01501383	Atherosclerosis, myocardial infarction, HIV infection, ischaemia–reperfusion injury	[341–346]
Monoclonal antibody	Anti-HMGB1 monoclonal antibody	Specifically neutralises extracellular HMGB1, blocks its binding to TLR4/RAGE receptor and inhibits NF-κB and MAPK signalling pathway activation	Preclinical	Sepsis, subarachnoid haemorrhage, neurodegenerative diseases, spinal cord injury	[348–357]
	SMU-CX24	TLR3 antagonist, inhibits the TLR3 signalling pathway and reduces oxLDL uptake by macrophages	Preclinical	atherosclerosis	[368]
	Vilobelimab (IFX-1)	Humanised IgG4 monoclonal antibody, selectively neutralises C5a molecules and blocks the C5a-C5aR1 signalling pathway, inhibiting neutrophil activation and excessive inflammatory response	Phase II/III NCT06701682 NCT05964413	severe sepsis	[358]
Diastase	Rasburicase	Uric acid oxidase, catalyses the conversion of uric acid into soluble allantoin, reducing blood uric acid levels	Listed/Phase IV NCT05312268 NCT00302653	Tumour lysis syndrome, acute hyperuricaemia	[360]
	Pegloticase	Polyethylene glycolated uric acid oxidase, extends half-life, reduces allergic reactions, catalyses uric acid breakdown	Listed/Phase II/III/IV NCT04772313 NCT03303989 NCT00675103	refractory gout	[360]
	DNase I	Degradation of extracellular DNA and NETs, reduction of immune complex deposition, inhibition of NF-κB pathway	Phase I/III NCT05453695 NCT01717742	Lupus Nephritis (LN)	[361, 440]
Small molecule inhibitors	TAK-242 (Resatorvid)	TLR4-specific inhibitor, blocks TLR4-MyD88 signalling, inhibits NF-κB and MAPK pathways, reduces pro-inflammatory cytokine release	Phase II/III NCT00143611 NCT00633477 NCT04620148	Sepsis, acute lung injury, cirrhotic liver failure	[363, 364, 440]
	Eritoran	TLR4 antagonist, blocks HMGB1-TLR4 axis, inhibits NF-κB pathway	Phase I/III NCT00334828 NCT00756912	sepsis	[367]
	FPS-ZM1	RAGE receptor inhibitor, blocks AGEs/RAGE binding and inhibits NF-κB p65 nuclear translocation and oxidative stress	preclinical	Alzheimer's disease, Parkinson's disease	[376–379]
	Azeliragon (TTP488)	RAGE receptor antagonist, blocks Aβ/RAGE binding, inhibits MAPK/ERK pathway, reduces Aβ oligomer deposition	Phase III NCT02080364	Alzheimer's disease	[380, 381]
	MCC950 (Canakinumab precursor)	NLRP3 inflammatory vesicle inhibitor, targets the NLRP3 NACHT structural domain and blocks ASC speckle formation and caspase-1 activation	Listed/Phase II NCT05964946 NCT00927810	CAPS, rheumatoid arthritis, cardiovascular disease, lung cancer prevention	[382–384]
	Tofacitinib	JAK1/JAK3 inhibitor, blocks IL-6/IFN-γ signalling pathway, inhibits DAMP downstream inflammatory cascade	Listed/Phase IV NCT06625450 NCT04267380 NCT02984020	Rheumatoid arthritis, ulcerative colitis, psoriasis	[387–392]
	RU.521	cGAS catalytic site inhibitor, occupies the cGAS active site, selectively inhibits dsDNA-dependent enzyme activity and reduces cGAMP production	Preclinical	Neuroinflammation, colitis, sepsis organ damage	[412]
	H-151	Covalent STING inhibitor, modifies STING C91 site, blocks palmitoylation and TBK1 interaction and inhibits type I interferon response	Preclinical	Acute kidney injury, amyotrophic lateral sclerosis, psoriasis	[418–420]
gene silencing technology	RNAi	RNAi technology, targeting mRNA and inducing its degradation to reduce receptor protein synthesis	Preclinical	Bladder cancer, sepsis	[429, 438]
	CRISPR-Cas9	CRISPR-Cas9 technology for targeted knockout and permanent silencing of target genes	Preclinical	Sepsis, peritonitis, type 2 diabetes, acute liver injury, Alzheimer's disease	[430–433, 439]

## Future challenges to targeted therapy with DAMPs and solutions to them

### Challenges

In traditional immunological research, the focus has been predominantly on the recognition of “non—self” antigens. However, an increasing body of evidence emphasizes the crucial role of endogenous danger signals in immune responses. DAMPs have emerged as significant biomarkers for disease progression and severity, and their involvement in numerous life—threatening aseptic inflammatory conditions has been well—documented. Over the past decade, although the mechanisms of DAMP production, their associated receptors, and downstream signaling pathways have been partially clarified, and although DAMP—targeted therapies have demonstrated potential in the treatment of inflammatory diseases and oncology, critical knowledge deficits still impede their clinical translation.

#### Complexity and diversity of DAMPs

Despite their potential therapeutic value, DAMP-targeted interventions face significant translational challenges. The most prominent among these challenges is the remarkable structural and functional heterogeneity within the DAMP-receptor system. Current research has identified more than ten distinct DAMPs, including ATP, HMGB1, S100 proteins, mitochondrial DNA, and hyaluronan fragments. Each of these DAMPs exhibits unique structural configurations that determine their diverse pathophysiological roles. Taking HMGB1 as a representative example, its redox state determines its differential immunomodulatory effects. The unoxidized form activates the NF-κB signaling pathway by binding to the TLR4, thereby stimulating the production of pro-inflammatory cytokines (e.g., TNF-α, IL-6) and amplifying immune responses. In contrast, oxidized HMGB1 undergoes conformational changes that enable it to bind to the RAGE. Subsequently, this binding suppresses pro-inflammatory signaling and, paradoxically, inhibits the production of anti-inflammatory cytokines. Upon oxidative modification, HMGB1 undergoes conformational rearrangement, which facilitates its binding to the RAGE. This redox-dependent interaction mediates immunosuppressive effects through two mechanisms: the suppression of pro-inflammatory signaling pathways and the induction of anti-inflammatory cytokine production [441].

DAMPs display notable heterogeneity in their expression across various diseases and even at different stages of the same disease, considerably escalating the difficulty of achieving precise regulation. For example, the NLRP3 inflammasome plays context-dependent roles. In the case of acute kidney injury, its moderate activation contributes to renal tissue repair by augmenting the clearance of damaged tubular epithelial cells and promoting the release of regenerative signaling molecules. Conversely, in chronic kidney disease, persistent endogenous stimuli (such as uric acid crystals and oxidative stress by—products) induce continuous activation of the NLRP3 inflammasome. This abnormal activation then triggers the proliferation of renal interstitial fibroblasts, excessive deposition of the extracellular matrix, and ultimately, progressive renal fibrosis. This functional duality is further manifested in diverse pathological conditions. In gouty arthritis, the intra—articular deposition of monosodium urate crystals activates the NLRP3 inflammasome, leading to the secretion of pro—inflammatory cytokines, including IL—1β, which mediate acute inflammatory responses. In contrast, during cutaneous wound healing, the degradation of hyaluronan generates low—molecular—weight fragments that activate TLR4 signaling pathways, subsequently promoting fibroblast proliferation and vascular endothelial cell migration to facilitate tissue regeneration. The determinants and environmental conditions that govern whether DAMP—induced inflammation results in beneficial tissue repair, harmful acute injury, or chronic inflammatory responses remain inadequately understood [323].

Furthermore, various DAMPs often interact within intricate signaling networks through shared receptor systems. A prominent instance is the NLRP3 inflammasome, which can be activated by multiple DAMPs, such as extracellular ATP, monosodium urate crystals, and mitochondrial DNA, among others. These results imply that monotherapeutic interventions targeting individual DAMPs are frequently insufficient for regulating the complex pathological networks involved. Moreover, while current clinical trials mainly concentrate on safety evaluations, there is a significant lack of rigorous validation of target engagement and pathway-specific therapeutic efficacy. Notably, DAMPs and PAMPs act in a synergistic manner to trigger inflammatory responses during pathogenic infections. Nevertheless, their respective contributions to inflammation induction are still poorly defined. Currently, the receptors targeting DAMPs are not only limited in diversity but also predominantly derived PRRs, which substantially hinders mechanistic investigations of both aseptic inflammation and the pathogenesis of inflammatory diseases.

#### Targeted delivery and pharmacokinetic limitations

The advancement of DAMP-targeted therapies encounters substantial pharmacological obstacles, especially in terms of target delivery and pharmacokinetic optimization, which considerably impede clinical translation. Empirical evidence suggests that certain DAMPs possess notably brief biological half—lives. A prime example is extracellular ATP, which undergoes swift enzymatic degradation by ectonucleotidases (notably CD39 and CD73) within minutes after entering the systemic circulation, resulting in a sharp decline in its concentration. This intrinsic metabolic instability significantly undermines the pharmacokinetic characteristics of conventional pharmacological agents aimed at ATP—mediated pathways, essentially constraining their therapeutic effectiveness. The rapid degradation of extracellular ATP by ubiquitous ectonucleotidases (CD39/CD73)—a phenomenon well—documented by Vultaggio—Poma et al.—represents a double—edged biological trait. While this inherent metabolic instability averts pathological signal over—amplification and receptor desensitization under physiological circumstances, it poses a fundamental pharmacokinetic hurdle for ATP—targeted therapeutic interventions [442].

Moreover, conventional targeted therapeutic agents, such as monoclonal antibodies and small—molecule inhibitors, encounter notable challenges in tissue penetration. In the tumor microenvironment, extracellular matrix components, including collagen and fibronectin, are highly cross—linked, jointly establishing a dense physical barrier. Considering the relatively high molecular weight of antibodies and the restricted diffusion ability of small—molecule inhibitors, these agents find it difficult to effectively penetrate this barrier. As a result, a large portion of the administered drugs accumulates near tumor cells without reaching the target site, thus significantly undermining the therapeutic efficacy of DAMP—targeted agents.

It is noteworthy that DAMPs are not only implicated in pathological states but also assume a crucial role in normal tissue repair processes. Taking HMGB1 as an illustration, this DAMP is released by damaged cells during wound healing and initiates a cascade of intricate cellular responses. These responses encompass promoting fibroblast proliferation and migration, as well as stimulating neovascularization by vascular endothelial cells, processes that are indispensable for tissue regeneration. Nevertheless, systemic administration of DAMP inhibitors results in extensive drug distribution, which may inadvertently interfere with the physiological functions of DAMPs in healthy tissues. For example, inhibition of HMGB1 may not only impede wound healing and compromise tissue regeneration but also disrupt immune-mediated pathogen clearance, thereby augmenting susceptibility to infections [443].

#### Clinical translation and individualized treatment bottlenecks

Precision medicine endeavors to identify and select optimal treatment strategies by taking into account individual variations in genetics, environment, and lifestyle. Although DAMP-targeted therapies have demonstrated potential in the treatment of inflammatory and immune-related diseases, their translation into clinical practice has been sluggish. The difficulty in predicting therapeutic efficacy represents a significant impediment. On one hand, the molecular diversity of DAMPs renders the pathological mechanisms in inflammatory responses intricate and variable. Currently, there is a dearth of reliable biomarkers capable of accurately reflecting treatment outcomes. On the other hand, the signaling pathways involving DAMPs possess complex regulatory mechanisms, such as cross-activation and positive/negative feedback. This complexity precludes the precise screening of the optimal patient population that can benefit from DAMP-targeted therapies, resulting in inconsistent clinical trial results. Some patients may experience poor treatment efficacy due to target mismatch or pathway compensation, while others may encounter risks of infection or disorders in tissue repair owing to excessive inhibition of physiological DAMPs. In addition, the substantial disparities in genetic backgrounds among patients and the high heterogeneity of disease manifestations lead to inconsistent treatment responses. Consequently, there is an imperative to develop personalized treatment regimens.

### Solutions

Despite the multifaceted challenges in targeting DAMPs and their receptors, their clinical translation continues to show significant promise.

#### Multi-target combination therapy

Multi—target therapeutic strategies have the potential to surmount the compensatory limitations of single—targeted therapies. This is achieved through the synergistic interference with critical DAMPs and their corresponding downstream signaling cascades, thereby attaining systemic control of the inflammatory cascade. For example, in sepsis, a positive feedback loop is formed between the HMGB1—TLR4 axis and the NLRP3 inflammasome, which amplifies the pro—inflammatory response. Simultaneous blockade of the HMGB1—TLR4 axis (e.g., via neutralizing antibodies) and the NLRP3 inflammasome (e.g., using MCC950) can decrease the secretion of IL—1β in experimental sepsis models. Moreover, it can notably alleviate damage to organs such as the lungs and kidneys. This approach exhibits superior efficacy when compared to single—therapy methods, thus emphasizing the advantages of synergistic multi—target inhibition [444].

In the realm of tumor therapy, the triple—combination therapy (NUAK1 inhibitor + statin + PD—1 antibody) developed by a research team for colorectal cancer serves as a representative instance. The NUAK1 inhibitor induces endoplasmic reticulum stress through the inhibition of the NRF2 antioxidant pathway, thereby triggering ICD of tumor cells and the release of DAMPs; e.g., CRT and ATP, DC—mediated antigen presentation. Nevertheless, this ICD process simultaneously activates a negative feedback loop via the mevalonate pathway. In this loop, the augmented cholesterol synthesis suppresses the accumulation of ROS, thus weakening the efficacy of ICD. Pharmacological inhibition of 3—hydroxy—3—methylglutaryl—coenzyme A reductase (HMGCR) with statins (e.g., simvastatin) reduces cholesterol levels, alleviates the mevalonate pathway—mediated suppression of ICD, and enhances the release of DAMPs, including HMGB1. Concurrently, the inhibition of the NLRP3 inflammasome mitigates IL—1β—driven immunosuppression within the tumor microenvironment, while PD—1 blockade further promotes CD8^+^ T—cell infiltration by surmounting T—cell checkpoint inhibition. This synergy of triple mechanisms significantly extends survival and elicits long—lasting anti—tumor immune memory in preclinical models [445].

These strategies indicate that multi—target combination therapy has shown the ability to conduct systematic interventions in complex pathological networks, whether in the inflammatory regulation of sepsis or the immune reconstruction of the tumor microenvironment. Looking ahead, the author posits that this research direction is anticipated to be further refined. By utilizing various emerging state—of—the—art technologies, it will be feasible to conduct in—depth analyses of the expression heterogeneity of DAMPs and their signaling pathways across different diseases and disease stages, thereby enabling the precise identification of intervention targets. Simultaneously, systematically exploring the synergistic mechanisms of multi—targets will contribute to surmounting the challenge presented by the complexity of DAMP signaling networks. With the continuous progress of research, multi—target combination therapy strategies are expected to realize their therapeutic potential in more domains, including fibrotic diseases, neurodegenerative diseases, and autoimmune diseases, offering novel perspectives and directions for the clinical intervention of complex diseases.

#### New drug delivery systems

The advancement of innovative drug delivery systems and formulations stands as a pivotal factor in revolutionizing therapeutic approaches. Progress in delivery vehicles, including nanoparticles and exosomes, is expected to optimize the pharmacokinetics of drugs and augment the targeted tissue enrichment of therapeutic agents, thus reducing systemic toxicity. Breakthroughs in biomaterials research have laid the groundwork for the development of intelligent nano—delivery systems [446]. In these systems, stimulus—responsive platforms, such as those responsive to pH [447], redox [448], and light [449] have been implemented for controlled drug release. For example, phenylboronic acid (PBA) functions as a pH—responsive ligand for the fabrication of stimuli—responsive polymers. A representative study conducted by Li et al. demonstrated that PBA—crosslinked nanoparticles could selectively release doxorubicin (Dox) in the acidic microenvironment of tumors, thereby enhancing therapeutic effectiveness and alleviating cardiotoxicity [450]. In the field of theranostics, Wang and colleagues engineered highly fluorescent cationic polyelectrolyte nanocarriers, which are capable of accurately tracing gene delivery pathways and simultaneously enhancing DNA transfection efficiency [451]. By integrating the fluorescent properties of the PLA—PEI copolymer with the pH—responsive characteristics of PBA, investigators have fabricated intelligent fluorescent boronate nanoparticles (FBNPs). These nanoparticles demonstrate structural stability under physiological circumstances and facilitate the rapid release of doxorubicin (Dox) within the acidic tumor microenvironment. Moreover, the near—infrared (NIR) fluorescence signals emitted by PLA—PEI enable the real—time monitoring of the spatial distribution of drug delivery, synergistically augmenting the therapeutic effectiveness [452].

Exosomes, as natural nanoscale extracellular vesicles, demonstrate distinctive advantages in the treatment of inflammatory and autoimmune diseases, attributed to their biocompatibility, low immunogenicity, and cross—barrier delivery capability. In sepsis models, exosomes derived from mesenchymal stem cells impede the activation of the NF—κB pathway through the delivery of anti—inflammatory molecules, including miR—155 and heme oxygenase—1 (HO—1). They also curtail the secretion of pro—inflammatory factors such as TNF—α and IL—6. Concurrently, they induce the polarization of M2 macrophages and the proliferation of Tregs, thereby enhancing the survival rates of mice and notably alleviating lung and kidney injury [453]. In the context of autoimmune diseases, exosomes exert therapeutic effects through the precise regulation of immune cell homeostasis. Exosomes derived from synovial mesenchymal stem cells (MSCs) of patients with RA, which exhibit high—level expression of miR—146a, can specifically target and IL—17—mediated pro—inflammatory signals and TLR pathways, thereby reducing the invasiveness of synovial fibroblasts [454]. Healthy mesenchymal stem cell-derived exosomes (MSC-Exo) safeguard chondrocytes against TNF-αinduced apoptosis through the delivery of the anti-apoptotic protein B-cell lymphoma 2 (Bcl-2), thereby retarding joint destruction [39]. Engineering modifications of exosomes can further enhance their therapeutic potential. Plant-derived exosome-like nanoparticles (PELNs) that deliver natural components, such as curcumin, can reduce the intestinal inflammation scores in ulcerative colitis mice through the regulation of the Th1/Th2 immune balance [455]. Engineered exosomes that are surface—modified with CD44 antibodies can specifically accumulate within inflamed joints, thereby augmenting the local concentration of methotrexate and substantially mitigating systemic side effects [456]. In the realm of clinical translation, a Phase I trial (NCT03898441) centered on MSC—Exo has verified its safety in the treatment of sepsis. This intervention is capable of reducing serum IL—6 levels and enhancing the organ failure scores of patients [457].

#### Immunomodulation combined with immunotherapy

Ultimately, the integration of immunomodulation and immunotherapy is anticipated to surface as a pivotal therapeutic strategy. Through the inhibition of the DAMP signaling pathway, it is feasible to reshape the tumor microenvironment and other pathological states, thus alleviating immunosuppression. Based on this approach, synergistic therapies, such as immune checkpoint blockade and adoptive cell therapy, have the potential to augment anti-tumor immune responses. This strategy may not only present novel prospects for cancer treatment but also offer a promising therapeutic route for inflammatory diseases, neurodegenerative disorders, and other arduous conditions.

In the integrated strategy of ICD induction and immunotherapy, nanoscale drug delivery systems (NDDSs) manifest remarkable advantages. Specifically, chemotherapeutic agents (e.g., doxorubicin, paclitaxel, oxaliplatin) delivered by NDDSs can effectively induce the release of DAMPs, such as CRT and HMGB1, thereby initiating ICD. Moreover, when combined with immune checkpoint inhibitors (ICIs), cytokines, or immunoadjuvants (e.g., CpG), NDDSs can notably enhance the recruitment of tumor-infiltrating lymphocytes and the activation of effector T cells, resulting in a more potent anti-tumor immune response [458–460]. Photodynamic therapy and photothermal therapy utilize nanocarriers to transport photosensitizers (e.g., IR780, zinc porphyrin) or photothermal agents (e.g., black phosphorus nanosheets). These therapeutic modalities can concurrently trigger tumor cell ablation via ROS generation or hyperthermia, while inducing immunogenic cell death. Significantly, when combined with immune checkpoint inhibitors or indoleamine 2,3-dioxygenase (IDO) inhibitors, this combinatorial strategy shows strong efficacy in suppressing both the progression of primary tumors and distant metastases, thus presenting remarkable potential for transforming immunologically "cold" tumors into states responsive to immunotherapy [461, 462]. Although radiotherapy-induced ICD is restricted by the immunosuppressive tumor microenvironment, its combination with immunotherapy effectively diminishes Treg infiltration, facilitates DC maturation, and substantially enhances the abscopal effects of radiotherapy [463, 464]. Multi—modality combinatorial therapy (e.g., chemotherapy—photodynamic therapy (PDT)—immune checkpoint blockade) facilitates the spatiotemporally regulated co—delivery of multiple therapeutic agents via precisely designed NDDSs. This approach not only augments therapeutic effectiveness but also alleviates the risk of resistance linked to monotherapies. For example, acid/enzymatic dual—responsive nanovesicles co—delivering oxaliplatin and photosensitizers, in combination with anti—CD47 antibody, can notably enhance tumor suppression rates and induce long—lasting immune memory [465].

The clinical translation of combination therapies based on NDDSs has achieved phased advancements. The phase III clinical trial of albumin-bound paclitaxel nanoparticles in combination with an anti-programmed death-ligand 1 (PD-L1) antibody for the treatment of metastatic triple-negative breast cancer has notably extended the survival period of patients with favorable safety profiles [466]. Nevertheless, the field continues to encounter numerous challenges. These encompass the optimization of the dosage and administration regimen of therapeutic agents, the enhancement of tumor models to more accurately mimic the human tumor microenvironment, the in—depth elucidation of the mechanism of ICD for the development of novel NDDSs, and the resolution of issues related to the quality control and large—scale production of NDDSs. Notwithstanding, with the continuous advancement of research and technological development, NDDS—mediated ICD combined immunotherapy holds promise in offering more efficacious personalized treatment alternatives for cancer therapy.

Moreover, precision medicine grounded in artificial intelligence and big data is also anticipated to reshape the treatment decision—making paradigm. AI algorithms can be employed to mine extensive clinical data, disease model data, and individual patient information for the accurate prediction of treatment responses and adverse reactions. Simultaneously, it can be integrated with real—time monitoring technology to dynamically track the treatment process, promptly adjust the drug dosage and combination regimen, and circumvent the latency and randomness of traditional treatments.

## Data Availability

Not applicable. All data was obtained from published studies and it is included in the manuscript.
